# Genetic Dissection of a Regionally Differentiated Network for Exploratory Behavior in *Drosophila* Larvae

**DOI:** 10.1016/j.cub.2015.03.023

**Published:** 2015-05-18

**Authors:** Jimena Berni

**Affiliations:** 1 Department of Zoology, University of Cambridge, Downing Street, CB2 3EJ Cambridge, UK

## Abstract

An efficient strategy to explore the environment for available resources involves the execution of random walks where straight line locomotion alternates with changes of direction. This strategy is highly conserved in the animal kingdom, from zooplankton to human hunter-gatherers [1–8]. *Drosophila* larvae execute a routine of this kind, performing straight line crawling interrupted at intervals by pause turns that halt crawling and redirect the trajectory of movement [9–11]. The execution of this routine depends solely on the activity of networks located in the thoracic and abdominal segments of the nervous system, while descending input from the brain serves to modify it in a context-dependent fashion [9]. I used a genetic method to investigate the location and function of the circuitry required for the different elements of exploratory crawling. By using the Slit-Robo axon guidance pathway to target neuronal midline crossing defects selectively to particular regions of the thoracic and abdominal networks, it has been possible to define at least three functions required for the performance of the exploratory routine: (1) symmetrical outputs in thoracic and abdominal segments that generate the crawls; (2) asymmetrical output that is uniquely initiated in the thoracic segments and generates the turns; and (3) an intermittent interruption to crawling that determines the time-dependent transition between crawls and turns.

## Highlights

• Exploration requires three neural functions: crawls, turns, and a transition switch
• Thoracic segments generate the asymmetric activity required for turns
• Crawls rely on symmetrical output from the thoracic and abdominal network
• Periodic pauses determine the frequency of transition between crawls and turns

## Graphical Abstract

**Figure:**
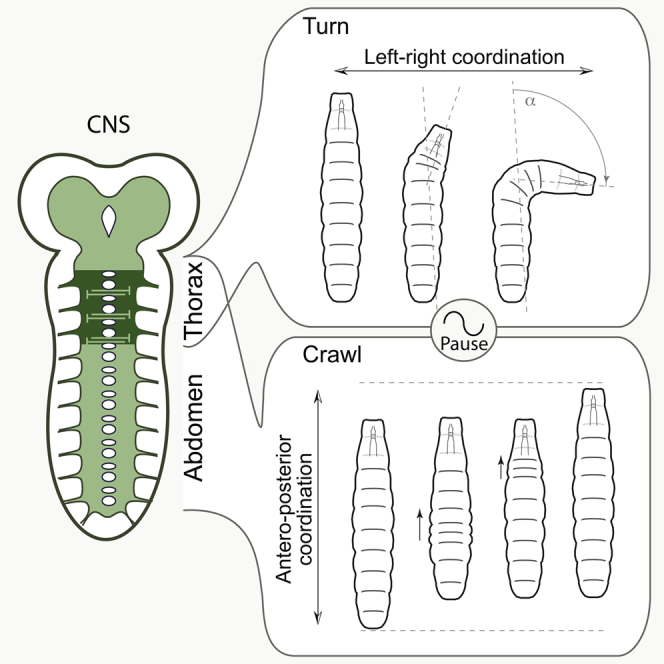

## Teaser

By genetically dissecting the neural circuitry of exploratory behavior in *Drosophila* larvae, Berni shows that the three elements of the routine, namely the crawl, the pause, and the turn, are separable and distinct and that each can be definitively assigned to different parts of a regionally specialized thoracic/abdominal neuronal network.

## Results

**Figure 1:**
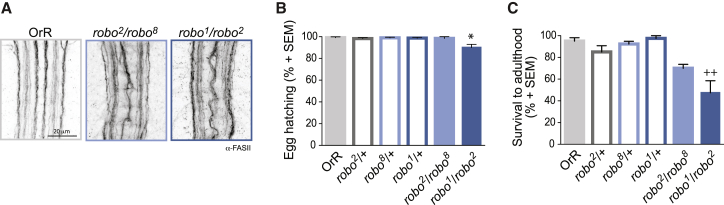
Survival of *Drosophila robo* Mutants (A) Staining showing the Fasciclin II (FASII) positive axon tracts in first instar larval nerve cords for the different allelic combinations used in the study. The increase in the number of axons that cross the midline produces the characteristic circular appearance around the commissures. The penetrance of this phenotype is complete (*robo*^*1*^/*robo*^*2*^ 82/82; *robo*^*2*^/*robo*^*8*^ 42/42 and [10]). See Figure S1 for evaluation of excitatory and inhibitory midline connectivity. (B) Average percentage of fertilized eggs hatching (±SEM). (C) Average percentage of hatched larvae that survived until emergence of the adult (±SEM). A Kruskal-Wallis test with Dunn’s multiple comparison comparing OrR with all genotypes and each allelic combination with their heterozygote alleles was performed. Asterisk (^∗^) indicates p < 0.05 when compared to OrR. ^++^p < 0.05 when compared to *robo*^*1*^/+.

Mutations in the gene *roundabout* (*robo*), coding for the receptor for the midline repellent Slit, cause axons and dendrites that will normally project on their own side of the CNS to cross the midline. This aberrant connectivity both of excitatory and inhibitory neurons leads to lethality at embryonic or larval stages (Figures 1A and S1; [10–12]). Interestingly, however, a high percentage of embryos with complete loss of function in the *robo* gene (*robo*^*1*^/*robo*^*2*^ and *robo*^*2*^/*robo*^*8*^) hatch, and these animals can be used for behavioral analysis (Figure 1 and [10–12]).

**Figure 2:**
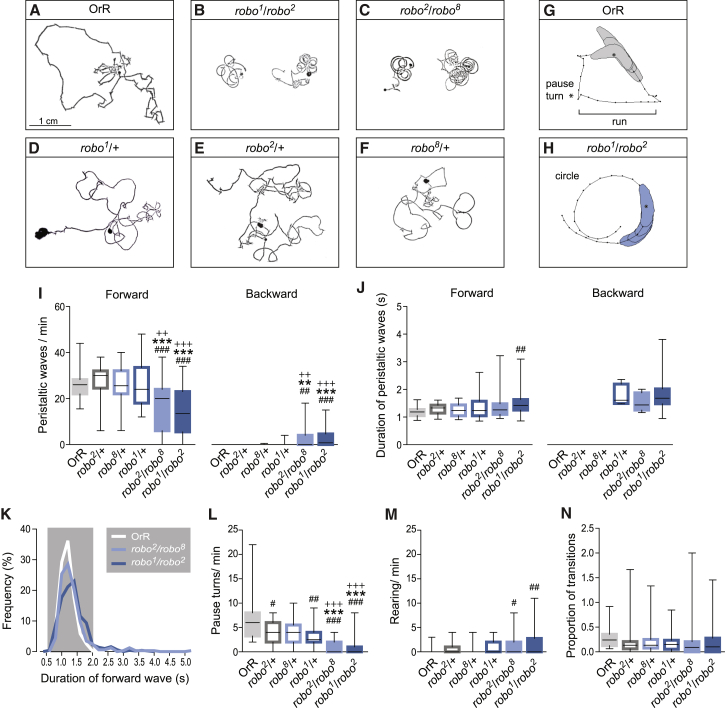
Locomotor Behavior of *robo* Mutant Larvae (A–F) Characteristic tracks of first instar larvae. OrR (A) and heterozygous *robo* mutant alleles (D–F) explore by alternating straight movements with turns. *robo* mutant larvae perform circular crawls (B and C). (G and H) Representative crawling patterns depicted by perimeter stacks. OrR larvae perform pause turns (G) (asterisk) by bending the anterior part of the body. *robo* mutants crawl in circles without performing turns (H). See also Movies S1, S2, S3, and S4 and Figure S2 for a detailed description of the behaviors. (I) Number of forward and backward waves per minute. (J) Duration of forward and backward waves in seconds. (K) Distribution of duration of forward peristalsis for all waves analyzed. The gray box highlights the duration of waves in OrR larvae. Binning is 200 ms; OrR n = 375; *robo*^*1*^/*robo*^*2*^ n = 471; *robo*^*2*^/*robo*^*8*^ n = 319. (L) Number of pause turns per minute. (M) Number of rearing events per minute. (N) Proportion of transitions. The number of pauses turns + rearing movements divided by the number of forward waves + backward waves was calculated. There are no significant differences between any genotype. A Kruskal-Wallis test with Dunn’s multiple comparison was used in (I), (J), (L), (M), and (N). Forward and backward waves were compared independently. Asterisk (^∗^) indicates comparison with *robo*^*2*^/+; + indicates comparison with the other heterozygote control; and # indicates comparison with OrR. ^∗^p < 0.05; ^∗∗^p < 0.01; ^∗∗∗^p < 0.001; ^+++^p < 0.001. 32–41 larvae were evaluated per group. Boundaries of boxplots represent first and third quartiles; the middle line indicates the median. Whiskers indicate the highest and lowest value of each experimental group.

In wild-type larvae, exploratory behavior consists of straight crawls, called runs, interrupted by pause turns [9, 13, 14]. The alternation between the two patterns of movements can be seen in the characteristic tracks left by wild-type newly hatched first instar larvae (Figures 2A and 2G; Movie S1). *robo*^*1*^/+, *robo*^*2*^/+, and *robo*^*8*^/+ heterozygote controls execute the same routine and produce the same pattern of tracks (Figures 2D–2F). On the other hand, *robo* mutants have abnormal exploratory behavior. Their tracks show that they are fully capable of performing extended forward crawls but that these runs follow a circular path without sharp redirections generated by pause turns, and as a consequence, *robo* mutant larvae remain within a limited region of the available substrate (Figures 2B, 2C, and 2H; Movie S2).

I quantified the crawling abilities of wild-type and heterozygous *robo* larvae and compared them with the crawling of larvae with mutant allelic combinations of *robo* (*robo*^*1*^/*robo*^*2*^ and *robo*^*2*^/*robo*^*8*^) by evaluating the denticle band movements (Figures 2I and S2). Wild-type and *robo* heterozygous larvae make equivalent numbers of forward waves of peristaltic contraction, but the *robo* mutants perform significantly less. In contrast, *robo* mutant larvae generate more backward waves (Figure 2I), although the total number of waves (forward and backward) is still significantly lower than controls. Notably, many *robo* mutant larvae have a postural deficit that causes them to lie on their sides rather than on their ventral surface with the consequence that the body is thrown into a curve generating circular paths instead of straight or slightly curved ones as observed during crawls in control larvae (Figures S2B and S2C). The duration of 96% of forward peristaltic waves in *robo*^*1*^/*robo*^*2*^ and 95% in *robo*^*2*^/*robo*^*8*^ mutant was the same as in wild-type animals (Figure 2K), although the distribution of the average duration per animal was only significantly different for *robo*^*1*^/*robo*^*2*^ null larvae (Figure 2J). Thus, even though the growth of axons and dendrites across the midline is highly abnormal in *robo* mutants, they are capable of generating waves of coordinated peristaltic crawling, and the execution of these forward waves is largely indistinguishable from those seen in controls.

Next, I evaluated the performance of pause turns. In *robo*^*1*^/*robo*^*2*^ and *robo*^*2*^/*robo*^*8*^, the number of pause turns is severely decreased, with a median value of zero indicating profound impairment (Figure 2L). This almost complete absence of pause turns (73% of the larvae analyzed for both genotypes never turned) is accompanied by a significant increase in the frequency of a movement similar to the previously described “rearing” behavior (Figures 2M and S2E; [9]).

The sequence of movement in rearing is very similar to a pause turn (Movie S3) with the significant difference that there is no unilateral contraction of muscles in the anterior segments producing a left- or right-hand turn (Figure S2E). Instead, during rearing, the larva pauses at the end of a forward wave of contraction and raises the anterior segments of the body as a consequence of a sequential and bilaterally symmetrical contraction of the thoracic segments. Then, as abdominal segment 1 (A1) contracts, the thoracic segments relax, and the anterior end of the animal is propelled downward to hit the substrate (Figure S2E; Movie S4). Larvae resume crawling after one or a series of such contraction and rearing movement cycles. The characteristic bilateral symmetry of the rearing phenotype suggests that the unilateral control of muscle contractions required to execute turns fails in *robo* mutants.

Interestingly, the intermittent interruption to crawling (the pause) occurs in all larvae irrespective of whether they are turning or rearing, and this indicates that the mechanism that underlies this periodic switch between patterns of movement is unaffected in *robo* mutants. To test this idea, I calculated the proportion of transitions defined as the number of turns plus rearings per number of waves (Figure 2N). There were no significant differences between controls and *robo* mutants. Thus, the probability of triggering a transition is not affected by the aberrations in midline crossing found in *robo* mutant larvae.

**Figure 3:**
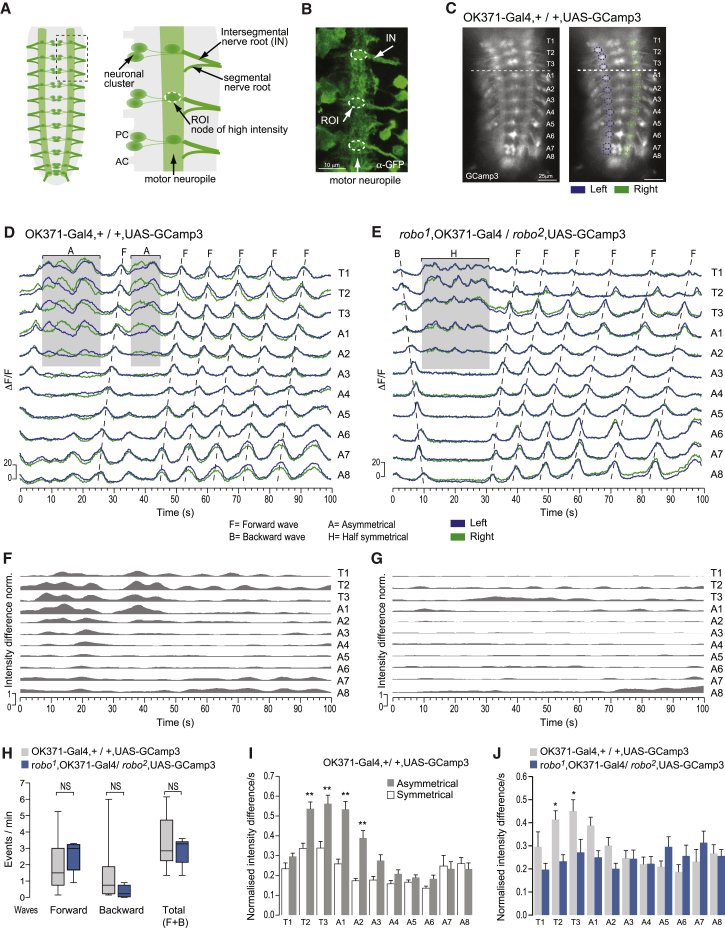
Output Activity of the CPG for Exploration (A) Schematic denoting the location in the motor neuropile of the anatomically distinct nodes of fluorescence at the level of the intersegmental nerve that has been analyzed: ROI, region of interest; PC, posterior commissure; AC, anterior commissure. (B) Equivalent region as in right panel of (A) in a CNS stained against GFP. (C) Snapshot of GCaMP3 fluorescence in glutamatergic neurons in an isolated nerve cord. Right: the ROIs on both sides of the nerve cord are shown. (D and E) Relative fluorescence change in isolated nervous systems. Left (blue) and right (green) sides of thoracic and abdominal segments were analyzed. Characteristic traces in control nervous system (D). Forward waves (“F”) of activity propagating along segments can be observed as well as asymmetric periods (“A”) in anterior segments. In *robo*^*2*^/*robo*^*1*^ mutants, forward and backward waves (“B”) are present (E). The asymmetric periods are absent, but symmetrical periods in the half anterior segments (“H”) can be observed. (F and G) Normalized intensity difference between the left and right side for the recording shown in (D) and (E), respectively. (H) Number of events per minute. n = 9 per group. A t test was performed comparing the number of waves between genotypes. Boxplots are described in the legend of Figure 2. (I and J) Average normalized intensity per second (±SEM). The periods of symmetrical and asymmetrical activity are compared in control animals showing that differences in activity occur mainly in anterior segments (I). An ANOVA (F_21,375_ = 18.36; p < 0.0001) with Bonferroni’s multiple comparison test comparing between genotypes for each segment was performed. ^∗∗^p < 0.001. n = 16 symmetrical periods and n = 20 asymmetrical periods. The difference of intensity for all active periods in control and *robo* mutants are compared (H). Anterior segments show a difference in symmetry. An ANOVA (F_21,161_ = 2.967; p < 0.0001) with Bonferroni’s multiple comparison test comparing between genotypes for each segment was performed. ^∗^p < 0.05. In *robo* mutant, there is no significant difference among segments, indicating that the output of the CPG is symmetrical (Bonferroni’s multiple comparison test comparing between segments). n = 8 per group. See also Figure S3 for a comparison between neuronal activity recorded with calcium imaging and behavior.

To show whether behavioral phenotypes of *robo* mutants arise from a defective output of the central pattern generators (CPGs) for exploration, independent of sensory input, I performed calcium imaging experiments on the isolated nervous system. The compact organization of the nervous system of the larva, where neuromeres are fused, lends itself to the simultaneous evaluation of spontaneous activity in all thoracic and abdominal segments. I used the OK371-Gal4 driver line for glutamatergic neurons [15] to target the genetically encoded calcium indicator UAS-Gcamp3 [16] to all motor neurons and quantified changes in signal intensity in a defined region of the neuropile at the level of the intersegmental nerve [17] on both sides of the nerve cord (Figures 3A–3C).

Forward and backward waves of calcium influx can readily be detected propagating along the abdominal and thoracic segments in nervous systems isolated from control animals (OK371-GAL4, +/+, UAS-GCamp3) (Figures 3D and 3H). These waves of activity are synchronous on both sides of the nervous system, and in a semi-intact preparation, they have been shown to coincide with the wave of muscular contraction [18], strongly suggesting that they are indeed equivalent to the output from the CPG for peristaltic waves. In the isolated nervous system, the intersegmental phases are the same as in crawling animals (Figure S3), while the speed of wave propagation is slower, as reported in semi-intact preparations [18, 19] and when sensory input was acutely removed in freely moving larvae [20], supporting the idea that the preparation is as healthy as possible.

In addition, bilaterally asymmetric patterns of calcium influx can be detected in the anterior segments of control larvae, and these appear to be equivalent to asymmetric activity associated with turns in intact animals (Figures 3D and S3). To quantify the asymmetric activity, I calculated the normal value of the difference in the signal intensity between the left and right sides for each segment (Figure 3F). A comparison of the values obtained during periods of asymmetric and symmetric activity in control CNSs shows that there is a significant difference in the thoracic T2 and T3 and abdominal A1 and A2 segments (Figure 3I), all of which are segments that contract unilaterally when a larva is turning.

Forward and backward waves of bilaterally symmetric calcium influx are also seen in nervous systems isolated from larvae mutant for *robo* at frequencies that are not significantly different from control nervous systems (Figures 3E and 3H).

In contrast to control nervous systems, however, the asymmetric periods are severely reduced in *robo* mutants, with only one nervous system showing an asymmetric period in the anterior segments (Figures 3E and 3G). At the same time, the number of symmetric periods initiated in the thoracic segments and propagating posteriorly as far as A5 is increased (Figure 3E).

To quantify the symmetry of activity, I performed a comparison among segments of the difference in the signal intensity between the left and right sides in *robo* mutant nervous systems, confirming the lack of asymmetry in the output of the CPG for exploration (Figure 3J). A further comparison of the average of activity between *robo* mutants and control animals (calculated for the entire recording) highlighted the thoracic T2 and T3 segments as the region generating the major difference of asymmetry (see Figures 3J and S2C for a correlation analysis).

These experiments confirm that there is no asymmetry to the output from the CPG of *robo* loss-of-function mutants and highlight the differential requirement for appropriate connectivity across the midline for the generation of turns as opposed to straight line peristaltic crawling. They also point to the thoracic segments as a region of the nervous system that may be essential for the generation of a turn.

In order to define more precisely the region of the nervous system where appropriate midline crossing is indispensable for the generation of asymmetric outputs, I dissected the *robo* behavioral phenotype by analyzing the performance of larvae with progressively more normal patterns of connectivity along the antero-posterior axis of the nervous system. I took advantage of the existence of a regulator of Robo, *commissureless*, that sequesters the receptor before it reaches the membrane and thereby generates a cell-autonomous *robo* mutant phenotype in the targeted cells [21, 22].

**Figure 4:**
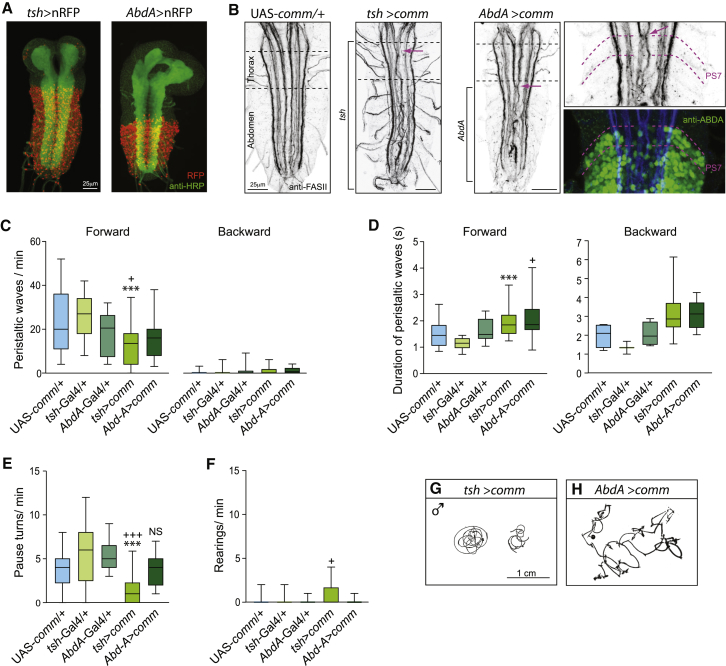
The Thoracic Segments Generate the Output for a Turn (A) Pattern of expression of the Gal4 lines used. The driver lines were crossed to the fluorescent reporter UAS-nuclear red fluorescent protein (nRFP). Anti-HRP staining was used to show the neuropile. *tsh*-Gal4 is expressed in the thoracic and abdominal segments [11]. In *AbdA*-Gal4, the *gal4* is inserted in the largest intron of the *abd-A* transcription unit and reproduces the expression profile of Abd-A [23, 24] from the posterior half of A1 until the posterior half of A7 [25]. (B) Midline crossing defect as depicted by staining against Fas II. *tsh*>*comm* have midline crossing defects in thoracic and abdominal neuromeres, while aberrant crossing is only present in the abdominal segments of *AbdA*>*comm* larval nervous systems starting in segment A1, coinciding with the anterior boundary of AbdA expression (right panels). Magenta arrows indicate the anterior boundary of midline crossing defects. (C) Number of forward and backward waves per minute. (D) Duration of forward and backward waves in seconds. (E) Number of pause turns per minute. (F) Number of rearing events per minute. (G and H) Representative tracks of male *tsh*>*comm* and *AbdA*>*comm* larvae. A Kruskal-Wallis test with Dunn’s multiple comparison was used. Asterisk (^∗^) indicates comparison with the same heterozygous driver line. ^∗^p < 0.05; ^∗∗∗^p < 0.001. + indicates comparison with UAS-*comm*/+. ^+^p < 0.05; ^++^p < 0.01; ^+++^p < 0.001. n_*UAS-comm*/+_ = 23; n_*tsh-Gal4*/+_ = 23; n_*AbdA-Gal4*/+_ = 14; n_*tsh>comm*_ = 30; n_*AbdA>comm*_ = 19. Boxplots are described in the legend of Figure 2.

Larvae that are UAS-*comm*; +/+; *tsh-*Gal4 (from now on *tsh>comm*) have normal patterns of connectivity in the brain lobes and subesophageal ganglion but have midline crossing defects in more posterior parts of the nerve cord, including all thoracic and abdominal segments (Figures 4A and 4B). The behavior of these larvae resembles that of *robo* mutant larvae (Figure 4). In particular, they crawl steadily and in a coordinated manner (Figures 4C and 4D), but the number of pause turns they make is significantly reduced, whereas rearing is increased compared to heterozygous controls (UAS-*comm*/+ and *tsh-*Gal4*/+*; Figures 4E–4G). The phenotype of *tsh>comm* animals is semi-penetrant; on average, they have the same defective performance of turns as both *robo* allelic combinations (non-significant differences between *tsh>comm*, *robo*^*2*^*/robo*^*1*^, and *robo*^*2*^*/robo*^*8*^ for rearings; Kruskal-Wallis and a Dunn’s multiple comparison, and pause turns, ANOVA with post hoc analysis with Bonferroni correction).

A striking difference appears in the behavior of larvae in which normal connectivity extends from the brain lobes through the subesophageal and thorax segments with a midline phenotype that begins in and extends caudally from the posterior compartment of abdominal segment A1 to A7 (UAS-*comm*;; +/+;; *AbdA-*Gal4, also *AbdA*>*comm*; Figures 4A and 4B). These animals are now completely normal in their performance of pause turns (Figures 4H); their number is not significantly different from the two heterozygous controls (UAS-*comm/+* and *AbdA-*Gal4/*+*) (Figure 4E). These animals also make very few rearing movements, and the number and duration of their forward and backward crawling waves are indistinguishable from those of the heterozygous controls (Figures 4C, 4D, and 4F).

In conclusion, these experiments define regional differences in a neuronal network for exploration. Posteriorly, bilaterally symmetric outputs required for a forward wave are initiated in the abdominal segments and pass forward to the thorax. More anteriorly, the asymmetric output required for unilateral contraction leading to a turn is initiated in the thorax and propagates to adjacent segments of the abdomen. While the propagation of bilaterally symmetric waves of contraction can proceed normally even if midline connectivity is disturbed, there is an absolute requirement for normal midline crossing for the asymmetric output of a turn, and it is this requirement that allows us to identify the thoracic segments of the network as the site of turn initiation.

## Discussion

In a previous paper, we showed that the exploratory crawling routine of the *Drosophila* larva is an intrinsic motor program, inherent to the thoracic and abdominal segments of the nervous system [9]. Using a genetic method, we were able to show that runs and pause turns continue normally if the brain and subesophageal ganglia are acutely silenced during exploratory crawling. The role of these more anterior segments of the nervous system is to modify the performance of the thoracic and abdominal routine in the presence of stimuli, for example, by altering the frequency and direction of turns when a food odor is detected [9, 26]. Here, I report the use of genetically targeted aberrations in axonal crossing at the midline to localize the regions of the nervous system that are essential for the facultative asymmetry in motor output that characterizes the turn as opposed to the symmetrical output of the straight crawl.

The first finding is that larvae that are null mutants for *robo* are capable of hatching and crawling over a substrate. Their crawling paths are unusually circular rather than straight, but this appears to be the effect of a postural deficit that causes the larvae to lie on their sides rather than on their ventral surfaces, with the consequence that the body is thrown into a curve, presumably by the differential strength of contraction in ventral as opposed to dorsal longitudinal muscles whose innervation is unaffected in *robo* mutants (Figure S4 and [27]). Despite this, the propagation of well-organized waves of muscle contraction from segment to segment proceeds normally during forward and backward crawls. Thus, the operation of the CPG for a straight crawl is unaffected by serious disruption to axonal crossing across the midline. The two sides of the animal are well coordinated, and this suggests that adequate and appropriate connections are maintained across the midline despite abnormal patterns of axonal growth. This is in stark contrast to the operation of the network required for turns. The performance of turns is completely abolished in the mutants, and this shows that, unlike the axial propagation of a wave, the generation of an asymmetric output depends absolutely on a normal pattern of connectivity across the midline. The simultaneous contraction of one side and relaxation of the other during a normal turn is likely to depend on the operation of reciprocal inhibitory connections across the midline [28] and one possible explanation is that as a consequence of the increased connectivity of both excitatory and inhibitory neurons across the midline (Figure S1), the strength of inhibition is decreased in comparison to excitatory connections in *robo* mutants. This notion is reinforced by the characteristic behavior that follows a pause in *robo* mutant larvae; contralateral inhibition is apparently overridden by symmetrical excitation so that the two sides of the animal contract together, causing the anterior segments to rear up, before relaxation and the resumption of a crawl.

The consequences of aberrant midline crossing for normally asymmetric motor outputs in the larva are reminiscent of the effects of induced defects in midline crossing seen in mice. In knockout mice for EphrinB3 or the EphA4 receptor tyrosine kinase, defective midline crossing of commissural interneurons [23, 29] causes synchronized activation of the normally reciprocating CPG for walking, with the result that the animals now exhibit a rabbit-like hopping phenotype, which is not unlike the rearing movement seen in *robo* mutant larvae. I find that an additional behavior in *Drosophila* larvae, which is likely to depend on reciprocal inhibition across the midline, namely self-righting from an inverted position, where asymmetric muscle contraction rotates the body, is also severely compromised in *robo* mutants (self-righting time: *robo*^*2*^/*robo*^*1*^ = 163 ± 23 s compared with *robo*^*2*^/+ = 22 ± 7 s, p < 0.001, and with *robo*^*1*^/+ = 36 ± 28 s, p < 0.01, Kruskal-Wallis test with Dunn’s multiple comparisons). These behavioral findings are reinforced by the observations of spontaneous activity in the isolated nervous system where fictive crawling-like behavior is signaled by bilaterally symmetric waves of calcium influx propagated forward and backward along the thoracic and abdominal nervous system. These symmetric waves are complemented by episodic, asymmetric, unilateral activity, which is confined to the thorax and most anterior abdominal segments and may represent the fictive equivalent of a turn. In *robo* mutants, symmetric wave-like events continue unabated in the isolated nervous system, but asymmetric activity is completely abolished.

The observation that turns and putative “fictive” turns are restricted to the anterior-most segments of the animal and the isolated nervous system prompted me to try to identify the specific parts of the nervous system required to initiate this episodic, asymmetric redirection of exploratory crawling. Since I had found that the turn is uniquely sensitive to aberrant midline crossing, I decided to use a genetic method to target these aberrations to specific segments of the nervous system. My results show that when crossing is disrupted in the thorax and the abdomen turns are abolished. However, when crossing is normal in the thorax but disrupted in the abdomen, the larvae perform a normal exploratory routine of runs and pause turns. Thus, I conclude that, although anterior abdominal as well as thoracic segments contract asymmetrically during a turn, the initiation of this asymmetric event is thoracic and that this asymmetry is propagated posteriorly through a descending pathway as the movement progresses. Thus, the thoracic part of the thoracic and abdominal network appears to have special characteristics that enable the generation and propagation of an asymmetric motor output. I find it interesting that, while the turn is blocked in *robo* mutants, the episodic interruption to crawling, the pause, which precedes the redirection of movement in the wild-type, is not. Although propagated, wave-like output is a property of the entire thoracic and abdominal network and turns are a property of the thorax, it is not clear which part of the system is responsible for the pause, or whether it depends on a fluctuating property of the whole network, such as the level of excitation. Elucidating the neuronal substrate and mechanism responsible for the time-dependent transition between crawls and turns will be essential to understand the dispersion characteristics of the larva both during spontaneous exploration and in response to sensory stimuli [9, 26, 30–34].

## Experimental Procedures

Materials and methods can be found in the Supplemental Experimental Procedures.

## Supplemental Information

Document S1. Supplemental Experimental Procedures and Figures S1–S4

Movie S1. Exploratory Behavior of a First Instar Control OrR Larva, Related to Figure 2

Movie S2. Exploratory Behavior of a First Instar *robo*^*1*^/*robo*^*2*^ Mutant Larva, Related to Figure 2

Movie S3. First Instar OrR Larva Executing a Pause Turn, Related to Figure 2

Movie S4. First Instar *robo*^*1*^/*robo*^*2*^ Larva Performing a Rearing Movement, Related to Figure 2

Document S2. Article plus Supplemental Information
