# Supplementary material for: Genetic Dissection of a Regionally Differentiated Network for Exploratory Behavior in Drosophila Larvae
Source: Curr Biol. 2015 May 18;25(10):1319–26. doi: 10.1016/j.cub.2015.03.023 (PMC4446794; doi:10.1016/j.cub.2015.03.023)
Supplement: Document S1. Supplemental Experimental Procedures and Figures S1–S4 [file mmc1.pdf]

Current Biology
Supplemental Information

Genetic Dissection of a Regionally Differentiated Network for Exploratory Behavior in *Drosophila* Larvae

Jimena Berni

Supplemental Figures

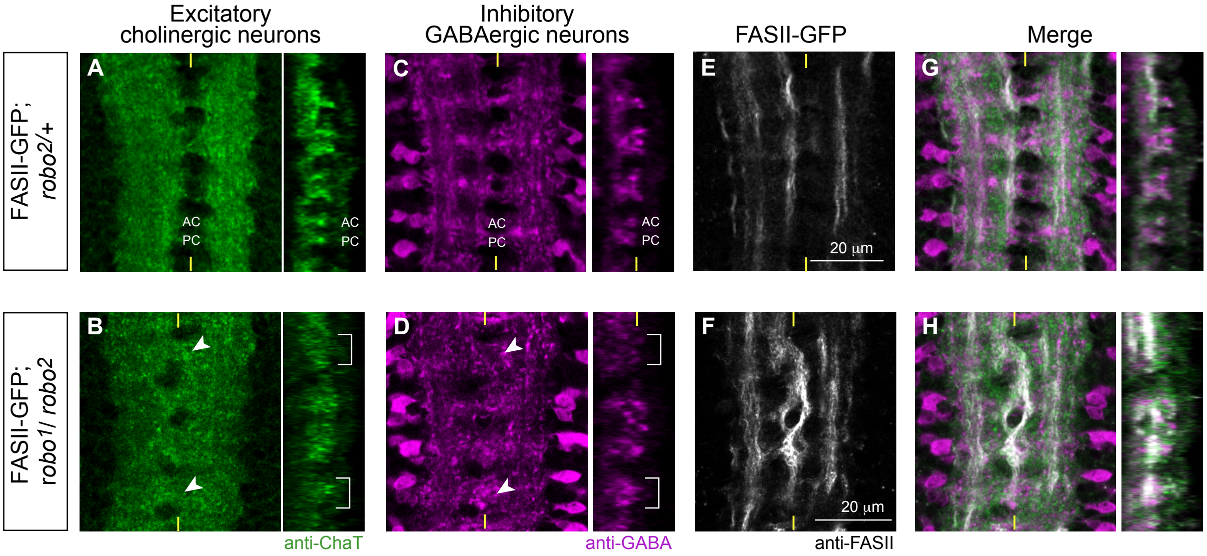

Figure S1 (related to Figure 1). Excitatory and Inhibitory Midline Connectivity is Increased in *robo* Null Mutants.

(A, B) Cholinergic excitatory neurons. In the control, FASII-GFP; $robo^2$/+, both the anterior commissures (AC) and posterior ones (PC) are clearly visible. In $robo^1$/ $robo^2$ mutants the commissures are thicker (arrows and brackets show some examples) due to the increased number of excitatory axons crossing the midline.

(C, D) GABAergic inhibitory neurons. The commissures in $robo^1$/$robo^2$ mutants are thickened as a consequence of increased inhibitory midline connectivity.

(E, F) FasII positive tracks for the XY slides shown, which is 1 μm thick.

(G,H) Merge images

Anterior is up. Yellow hash marks indicate the positions of the XZ section.

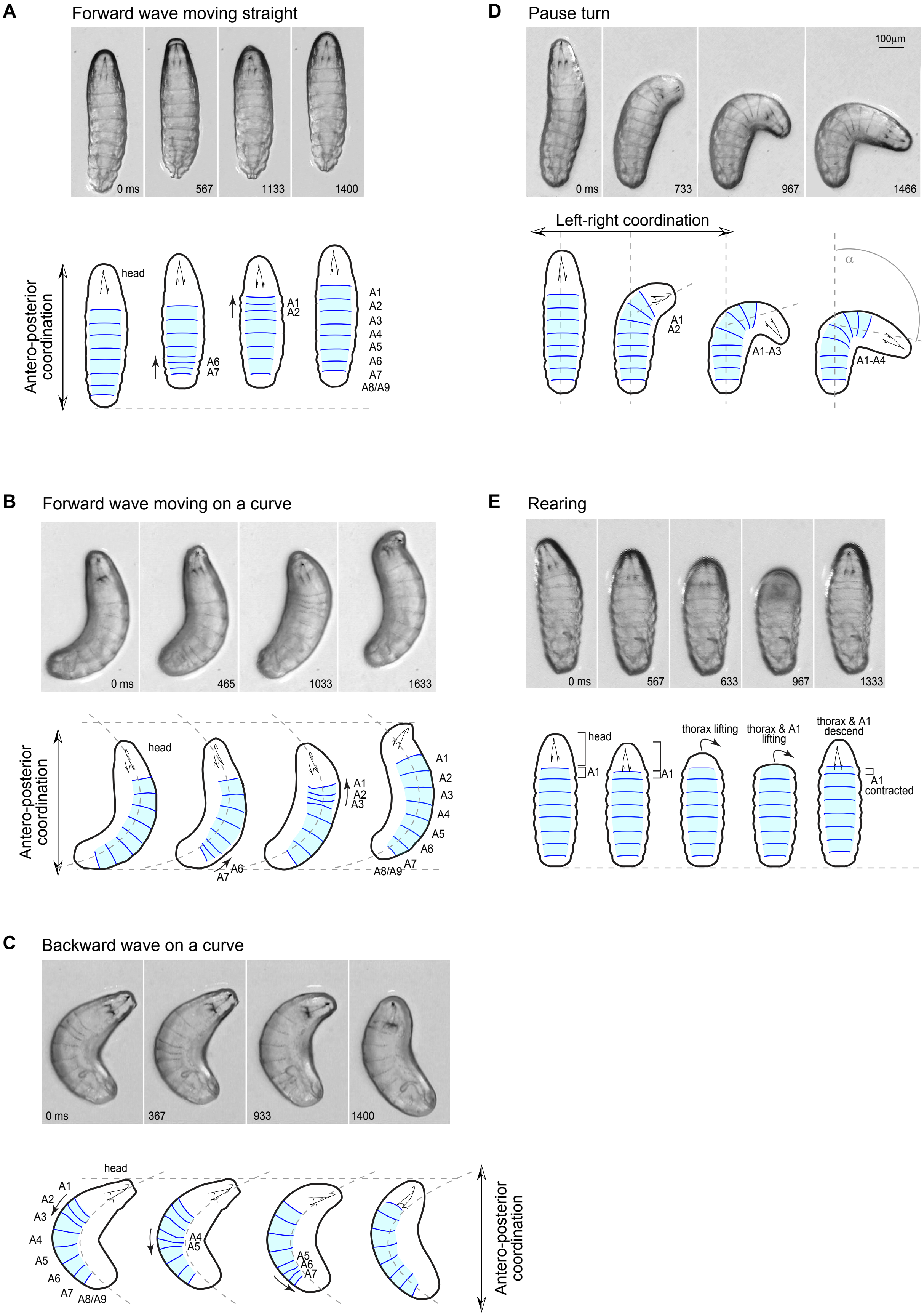
A
Forward wave moving straight
0 ms
567
1133
1400
Antero-posterior coordination
head
A1
A2
A6
A7
A1
A2
A3
A4
A5
A6
A7
A8/A9
B
Forward wave moving on a curve
0 ms
465
1033
1633
Antero-posterior coordination
head
A6
A7
A1
A2
A3
A1
A2
A3
A4
A5
A6
A7
A8/A9
C
Backward wave on a curve
0 ms
367
933
1400
head
A1
A2
A3
A4
A5
A6
A7
A8/A9
A4
A5
A5
A6
A7
Antero-posterior coordination
D
Pause turn
100µm
0 ms
733
967
1466
Left-right coordination
α
A1
A2
A1-A3
A1-A4
E
Rearing
0 ms
567
633
967
1333
head
A1
A1
thorax lifting
thorax & A1 lifting
thorax & A1 descend
A1 contracted

Figure S2 (related to Figure 2). Description of Larval Patterns of Movement During Exploration.

Time-lapse of single peristaltic waves (A, B, C), a pause turn (D) and a rearing (E) with their corresponding diagrams below.

(A) Forward wave from a wild type. The larva advances in a straight line. Note that *robo* null mutant also perform forward waves in a straight line.

(B) Forward wave and backward wave (C) from a *robo*^{1}/*robo*^{2} mutant lying on one side. The larva is thrown into a curve.

(D) A pause turn from a wild type larva

(E) Rearing behaviour of a *robo*^{1}/*robo*^{2} mutant.

Arrows indicate the contracting abdominal segments at each time-point during a forward or backward peristaltic wave. The numbered abdominal segments in (B) correspond to the unilaterally contracting segments at each time-point during a turn. The light blue area indicates the denticle bands area in contact with the substrate.

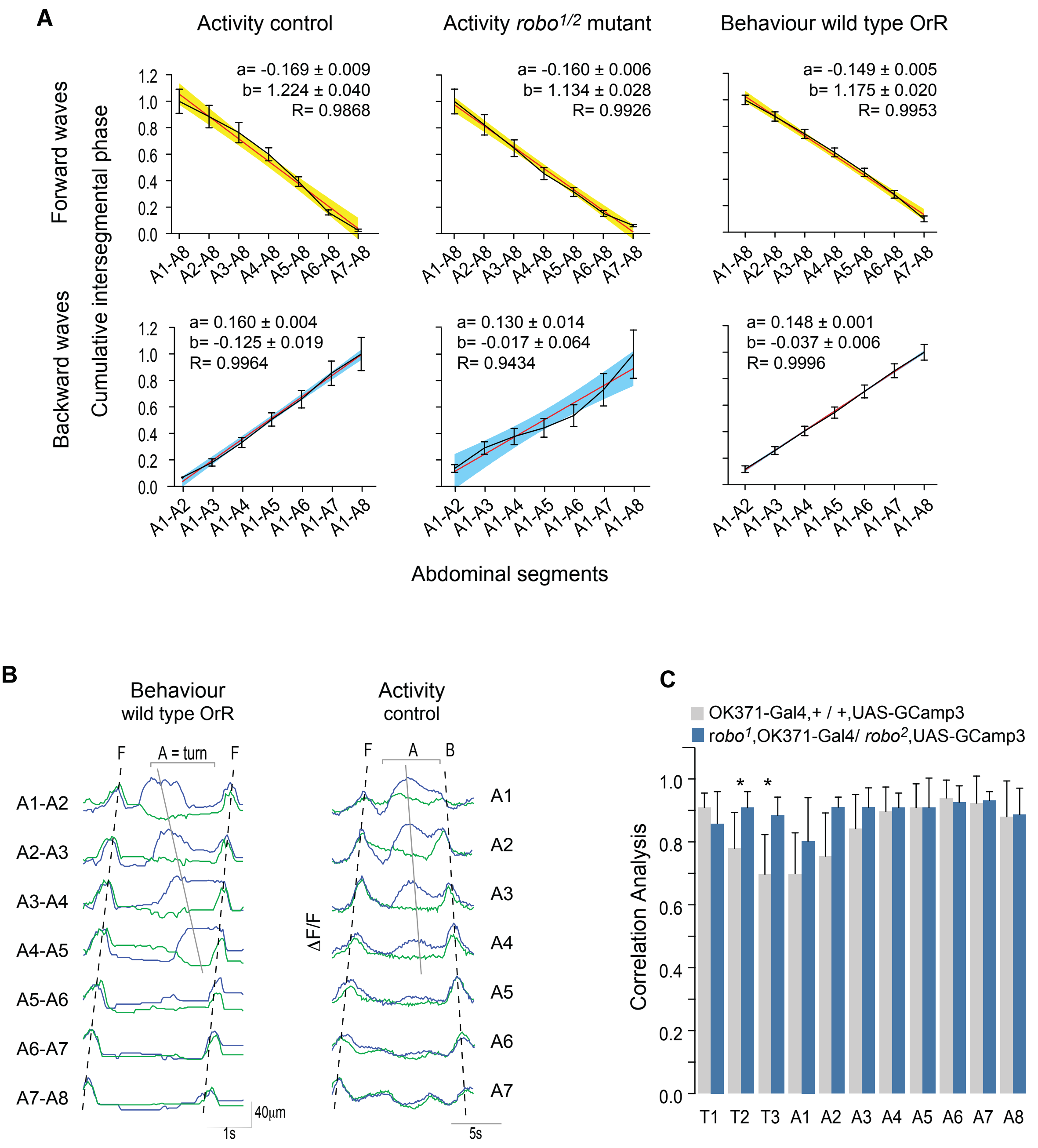

Figure S3 (related to Figure 3). Comparison between Neuronal Activity Recorded with Calcium Imaging and Behaviour.

(A) Comparison of intersegmental phases in isolated nervous system and crawling animals. Average cumulative intersegmental phase (+ SEM) in isolated nervous system and crawling larvae. The average phase of activity or contraction for each segment has been calculated respect to the beginning of the wave and is therefore refers to "cumulative". Control larvae very rarely perform backward crawling. A gentle poke in the front of the animals was applied to stimulate them to do so. A linear regression analysis was performed, red line. The confidence intervals are highlighted in yellow and blue. a= slope; b= y value at x=0. The slopes of forward waves are not significantly different while the intercept at x=0 are (F =

9.03; DFn=2 DFd=17; P=0.002). There are no significant differences amongst backward waves.
Number of waves (Number of animals): Forward: control= 54 (9); *robo*$^{1/2}$= 66 (9); behaviour= 35 (15). Backward: control= 24 (9); *robo*$^{1/2}$= 10 (9); behaviour 14 (9).
(B) Comparison of the turning behaviour and asymmetrical activity in isolated nervous system. The temporal dynamics of segment activity and segment contraction during the asymmetrical period "A" are similar. A backward wave initiates only on one side and propagates posteriorly as far as A5.
The complete genotype of control animals is OK371-Gal4,+/ +,UAS-GCamp3 and of *robo*$^{1/2}$ animals is *robo*^{1}, OK371-Gal4 / *robo*^{2} ,UAS-GCamp3; F means forward wave and B means backward wave.
(C) A correlation analysis (average $\pm$ SEM) for the entire calcium imaging recordings was performed**.** Anterior segments T2 and T3 show a difference in symmetry amongst genotypes. An ANOVA comparing the r values between genotypes ($F_{21,161}$ = 2.386; p = 0.001) with post hoc comparison was performed. * p < 0.05. n = 8 per group.

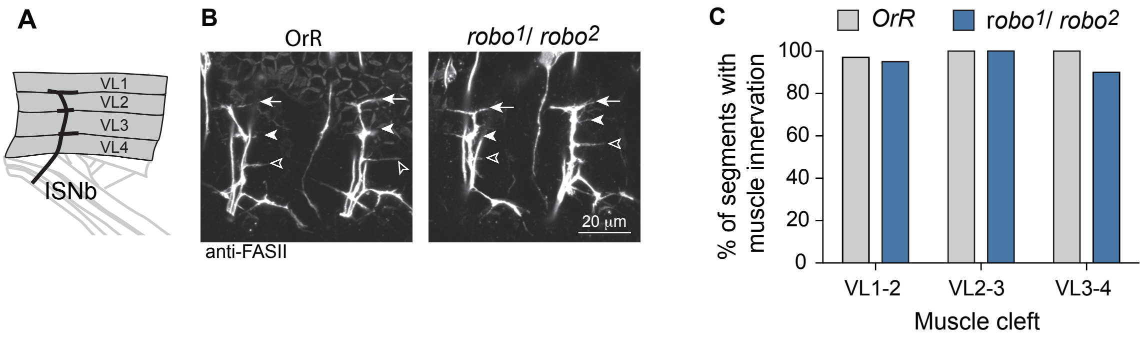

Figure S4 (related to the Discussion). The Innervation to the Ventral Longitudinal (VL) Muscles is Not Affected In *robo* Mutants.

(A) Projections of intersegmental nerve b (ISNb) to the embryonic ventral longitudinal muscles VL1 (12), VL2 (13), VL3 (6) and VL4 (7).

(B) ISNb motor neuron visualised with FasII antibody in stage 17. Arrows point to the VL1-2 innervation, filled arrowhead to VL2-3 and empty arrowhead to VL3-VL4.

(C) Percentage of segments showing innervation to VL muscles. An exact fisher test was performed comparing the wild type OrR with $robo^1$/ $robo^2$ mutants for each muscle cleft. VL1-2 P=1; VL2-3 P=1 and VL3-4 P=0.1153. 39 segments were analysed for each genotype.

Supplemental Experimental Procedures

Exploratory behaviour. Eggs were collected from flies kept on apple juice agar plates supplemented with yeast paste at 25°C.

Newly hatched first instar larvae were transferred to a 5cm petri dish coated with 0.9% agarose. The plate was inverted to view the denticle bands and 30 s – 2 min movies were captured at 30 fps with a JVC TKC1380 camera mounted on a Leica M420 microscope at 32X magnification. The wave duration was calculated as in Gjorgjieva *et al.* 2013 [S1]. The duration of a forward peristaltic wave was defined as the time between the first movement of the posterior end of the larva and the last movement of the anterior band of the first abdominal segment (A1). The reverse was used for backward waves (from A1 until the posterior end of the larva). The movements of thoracic segments were not included since their denticle bands are almost invisible and they move more independently. The timing of contraction was quantified with the open source software VCode 1.2.1 (http://social.cs.uiuc.edu/projects/vcode.html). Turns were defined based on the detailed pattern of movements that can be evaluated by observing the denticle bands, they consist of an initial pause followed by the unilateral backward contraction initiated in thoracic segments and propagating backward until A4-A5. The different animals were analyzed blind.

The duration and number of waves or turns performed by each animal was averaged and represent one data, except in Figure 2K (see legend & results). At least 3 independent experiments were performed.

To image the tracks , newly hatched larvae were allowed to crawl for 3 hours at 25 degrees in the dark. The plates were inverted on top of an Ilford speed 3.1 m 100 photographic paper and illuminated for 4 seconds using an enlarger. Standard photographic method for development was used: 2 mins in Kodak Professional developer D19, rinse in water, 2 mins in Ilford Hypan rapid fixer, thorough wash in water. Black and white were then digitally inverted and background removed (technique developed by Michael Bate).

Dynamic Image Analysis (DIAS) software 3.4.2 (Sholl technologies, USA) was used to generate the perimeter stacks in Figures 2G and 2H.

Survival experiments. Hatching: adults were allowed to lay eggs overnight. The total number of larvae hatching from fertilized eggs was counted. Adult survival: 20 first instar larvae of each genotype were transferred and grown on apple juice agar plates supplemented with yeast paste. The number of adult emerging was counted. Experiments were repeated at least 3 times. Allelic combinations of $robo^1$, $robo^2$ and $robo^8$ were used to evaluate the *robo* loss of function in homozygosis while keeping in heterozygosis any other possible unspecific mutations present in the stocks that were generated with the mutagen ethyl methanesulfonate (EMS) [S2].

Calcium imaging. Experiments were performed on first instar larvae, 0– 4 h after hatching, at room temperature (23-25°C). Larvae were washed in H_2O and their nervous systems dissected in external saline for electrophysiology that consisted of: 135 mM NaCl, 5 mM KCl, 4 mM MgCl2.6H2O, 2 mM CaCl2, 5 mM N Tris (hydroxymethyl) methyl-2-aminoethanesulfonic acid and 36 mM sucrose, pH 7.15 [S3]. The isolated nervous systems were then stabilized by adhesion to a polylysine-coated cover slip glued to a 5 cm petridish filled with external saline. Preparations with a drift were excluded from the analysis.

A Leica DML-FS microscope with a 40x dipping objective was used for imaging. A 488 ± 15 nm wavelength light produced by a Cairn Optoscan Monochromator and passed through the epifluorescence port of the microscope was used to excite GCamp3. 2000 frames movies were captured using a cooled EMCCD Andor iXon DV887 camera at 5Hz at 512x512 pixel resolution using 2x pixel binning, and 2x frame binning through time. Data were transferred to Andor IQ software (version 1.9.1), recorded to a kinetic image disc.

Intensity information was extracted on ImageJ from regions of interest (ROIs). ROIs were defined as an oval enclosing the area of maximum intensity in the motor neuropile at the intersegmental nerve root [S4-S5] in abdominal segment one (A1). This was copied for all the other segments on the right and left side of the nerve cord. $\Delta F/F = (f_n - f_0)/f_0 * 100$ where f_0 is the average intensity.

Difference of Intensity between sides was calculated as: abs[$\Delta F/F$ left- $\Delta F/F$ right]. The area below curves filtered with a 2s window were calculated using spike 2. The average $\Delta F/F$ was normalized among experiments. Silent periods were not included in the average of normalized intensity. A correlation analysis was also performed using the entire recording.

The number of forward and backward events was empirically determined evaluating the $\Delta F/F$ curves along the abdominal segments.

Immunohistochemistry. Nervous systems of newly hatched first instar larvae were dissected in PB (100mM NaH2PO4/Na2HPO4) pH 7.2, transferred to a polylysine-coated cover slip fixed with 4% formaldehyde in PB for 20 min at room temperature (RT) and rinsed in PBS plus 0.3 % Triton X-100 (PBT) 3 X 15 min. Specimens were then incubated with anti-Fas II ID4 1/10; anti-ChaT 4B1 1/20 (Developmental Studies Hybridoma Bank, USA); rabbit anti-GABA A2052 1/2000 (Sigma) or chicken anti-GFP 1/2000 (Abcam) in PBT overnight at 4°C in a wet chamber, washed in PBT 4 X 15 min, and incubated with secondary antibodies at 1/500 in PBT for 3 h at RT: Alexa568 anti-Mouse; CF633 anti-rabbit; Alexa488 anti-Chicken (Invitrogen) and fluorescence-conjugated anti-HRP (Jackson Immuno Labs) at 1:50. Secondary antibodies were washed 4 X 30 min in PBT and specimens were mounted in Vectashield (Vector Laboratories) between two aluminium-foil spacers, to avoid distortion of nerve cords, under number 1 cover glasses. Image stacks were captured on a Leica TCS-SP-5 confocal microscope.

Embryo fixation and staining were performed as described in Patel 1994 [S6], anti-Fas II ID4 1/5.

Fly stocks. The following fly lines were provided by Bloomington Stock Center: UAS-stinger-RFP and Oregon-R. OK371-Gal4 is expressed in glutamatergic neurons, which includes all motor neurons and a subset of interneurons [S7]. UAS-*commissureless* on X [S8]. *AbdA*-gal4 was a present of Samir Merabet [S9]. *robo*^{1}, *robo*^{2}, *robo*^{8} were kindly shared with me by Guy Tear. *tsh*-Gal4 was described in [S10]

Statistical Analysis. Statistical analysis was performed employing the Prism Graphpad 5.0b software package (2009). Normality was tested with a Kolmogorov-Smirnov test with Dallal-Wilkinson-Lillie for *p* value. A Bartlett's test for equal variances was also performed before deciding if a parametric or non-parametric test was going to be used to analyse the data.

Supplemental References

S1. Gjorgjieva, J., Berni, J., Evers, J. F., and Eglen, S. J. (2013). Neural circuits for peristaltic wave propagation in crawling *Drosophila* larvae: analysis and modeling. Front Comput Neurosci *7*, 24.

S2. Seeger, M., Tear, G., Ferres-Marco, D., and Goodman, C. S. (1993). Mutations affecting growth cone guidance in *Drosophila*: genes necessary for guidance toward or away from the midline. Neuron *10*, 409–426.

S3. Muraro, N. I., Weston, A. J., Gerber, A. P., Luschnig, S., Moffat, K. G., and Baines, R. A. (2008). Pumilio binds para mRNA and requires Nanos and Brat to regulate sodium current in *Drosophila* motoneurons. J Neurosci *28*, 2099–2109.

S4. Landgraf, M., Bossing, T., Technau, G., and Bate, M. (1997). The origin, location, and projections of the embryonic abdominal motorneurons of *Drosophila*. J Neurosci *17*, 9642–9655.

S5. Kohsaka, H., Takasu, E., Morimoto, T., and Nose, A. (2014). A group of segmental premotor interneurons regulates the speed of axial locomotion in *Drosophila* larvae. Curr Biol *24*, 2632–2642.

S6. Patel, N. H. (1994). Imaging neuronal subsets and other cell types in whole-mount *Drosophila* embryos and larvae using antibody probes. Methods Cell Biol. *44*, 445–487.

S7. Mahr, A., and Aberle, H. (2006). The expression pattern of the *Drosophila* vesicular glutamate transporter: a marker protein for motoneurons and glutamatergic centers in the brain. Gene Expr Patterns *6*, 299–309.

S8. Kidd, T., Russell, C., Goodman, C. S., and Tear, G. (1998). Dosage-sensitive and complementary functions of *roundabout* and *commissureless* control axon crossing of the CNS midline. Neuron *20*, 25–33.

S9. Hudry, B., Viala, S., Graba, Y., and Merabet, S. (2011). Visualization of protein interactions in living *Drosophila* embryos by the bimolecularfluorescence complementation assay. BMC biology *9*, 5.

S10. Fasano, L., Röder, L., Coré, N., Alexandre, E., Vola, C., Jacq, B., and Kerridge, S. (1991). The gene teashirt is required for the development of *Drosophila* embryonic trunk segments and encodes a protein with widely spaced zinc finger motifs. Cell *64*, 63–79.
