# Supplementary figures and images for: Genetic Dissection of a Regionally Differentiated Network for Exploratory Behavior in Drosophila Larvae
Source: Curr Biol. 2015 May 18;25(10):1319–26. doi: 10.1016/j.cub.2015.03.023 (PMC4446794; doi:10.1016/j.cub.2015.03.023)

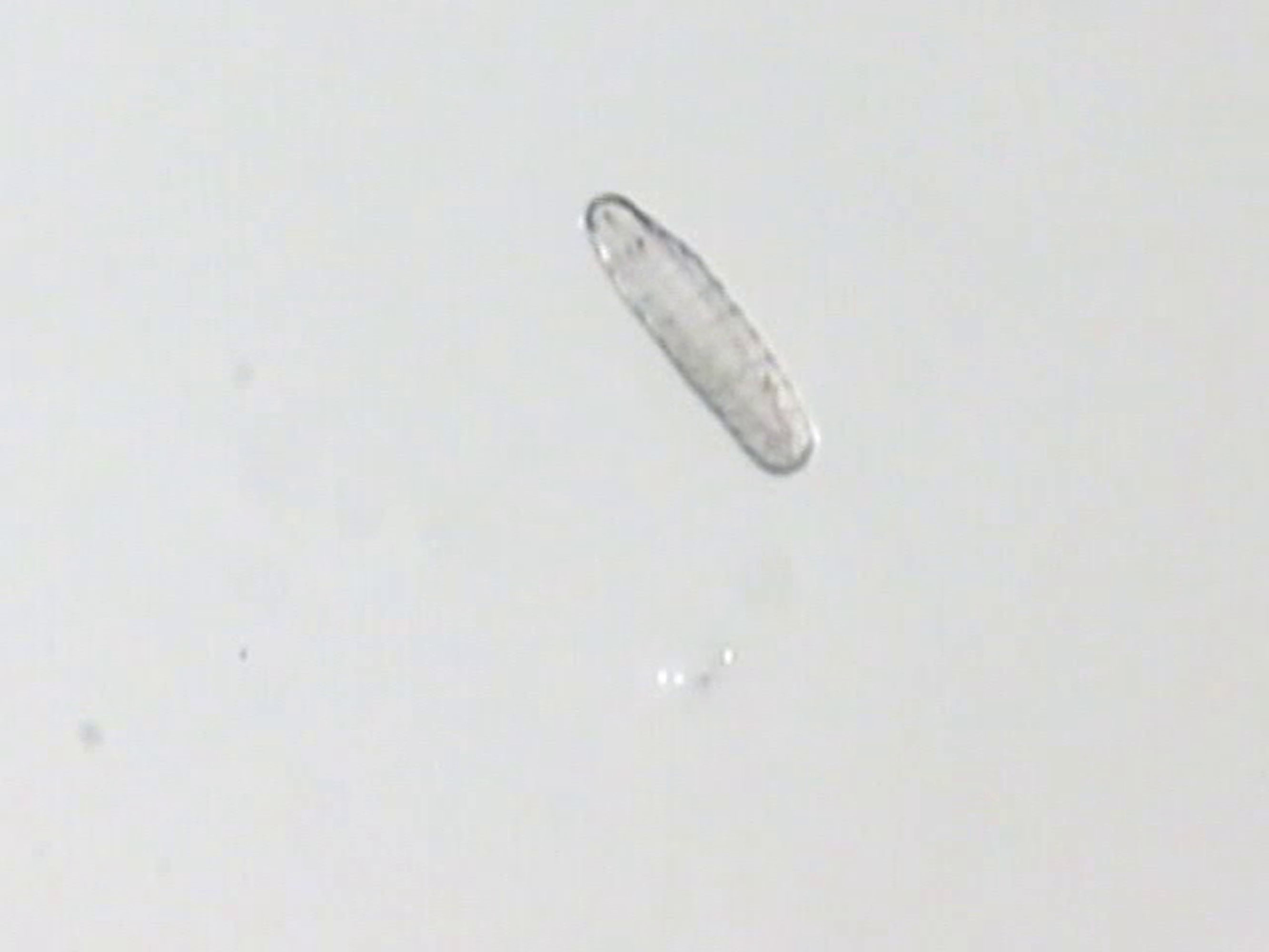

Supplement: Movie S1. Exploratory Behavior of a First Instar Control OrR Larva, Related to Figure 2 [file mmc2.jpg]

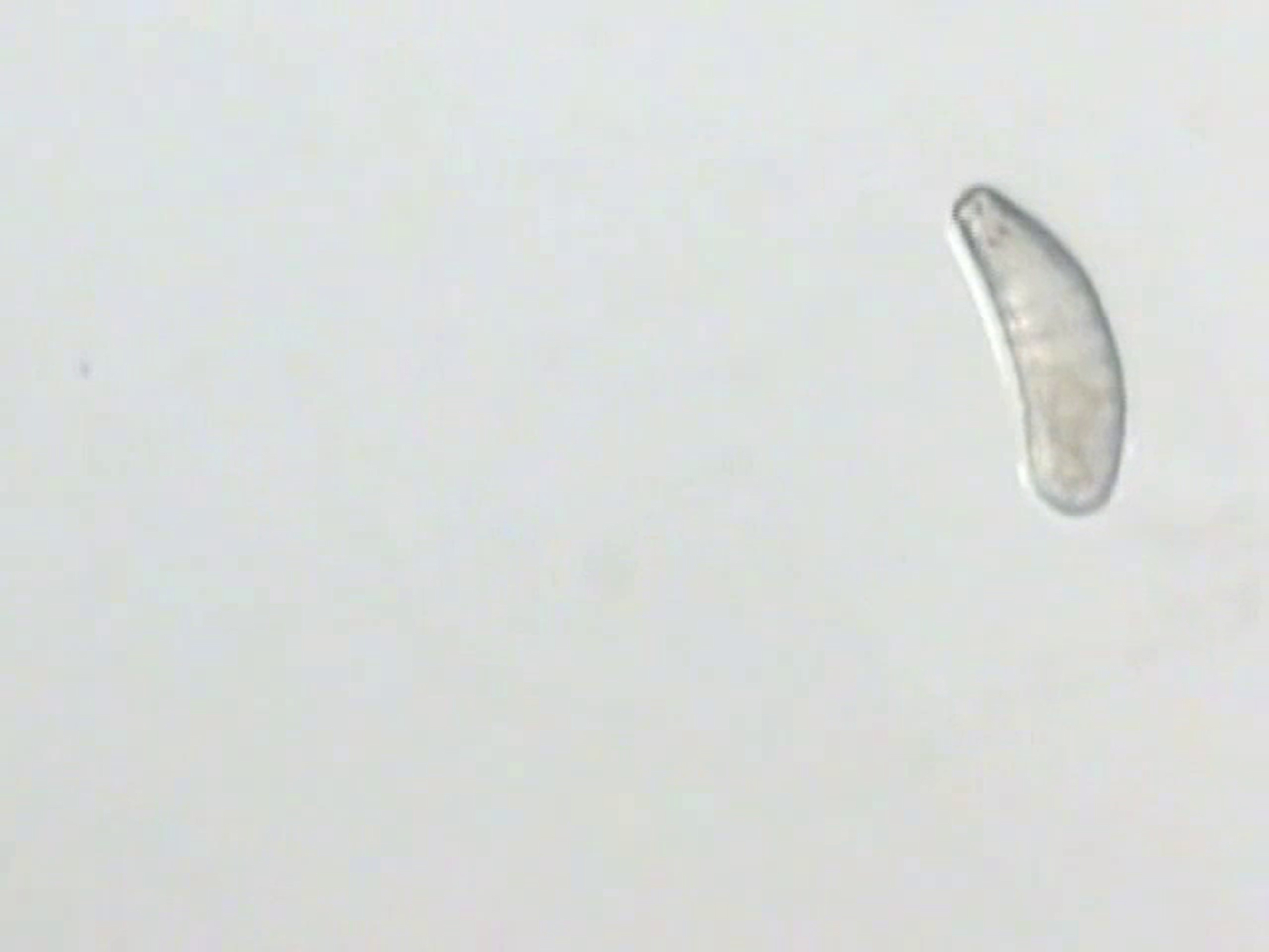

Supplement: Movie S2. Exploratory Behavior of a First Instar robo1/robo2 Mutant Larva, Related to Figure 2 [file mmc3.jpg]

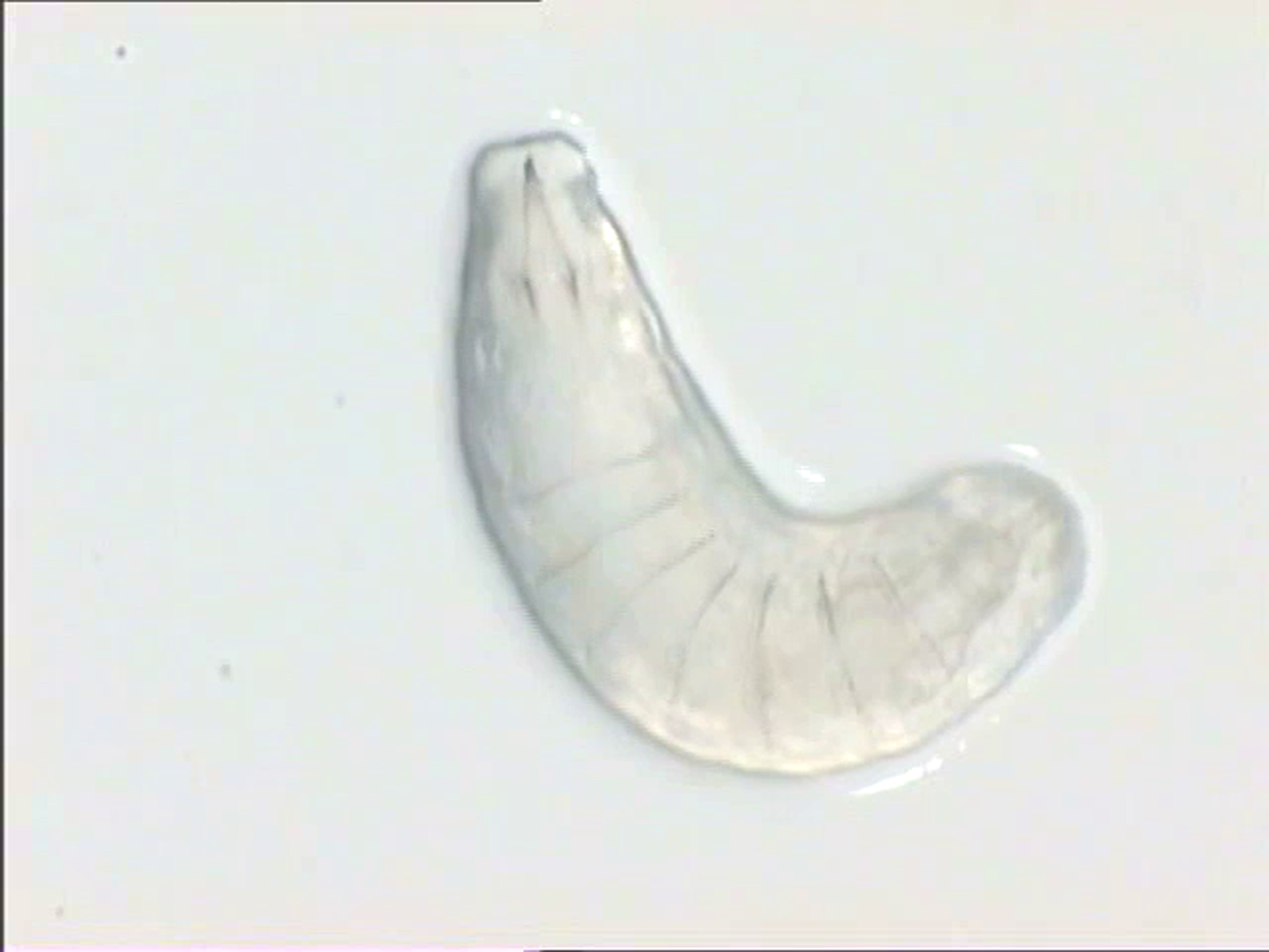

Supplement: Movie S3. First Instar OrR Larva Executing a Pause Turn, Related to Figure 2 [file mmc4.jpg]

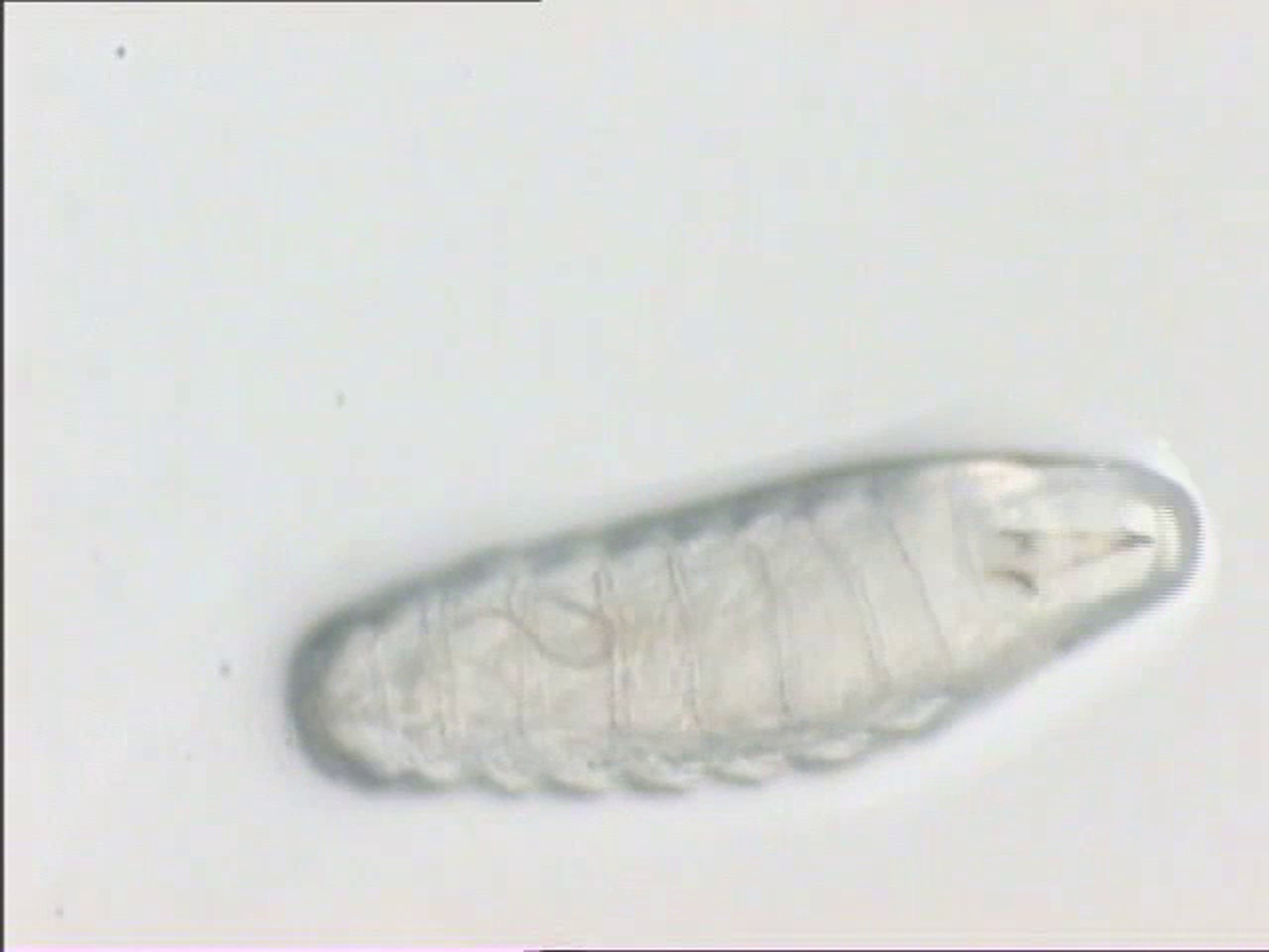

Supplement: Movie S4. First Instar robo1/robo2 Larva Performing a Rearing Movement, Related to Figure 2 [file mmc5.jpg]
