# Supplementary material for: Genetic Dissection of a Regionally Differentiated Network for Exploratory Behavior in Drosophila Larvae
Source: Curr Biol. 2015 May 18;25(10):1319–26. doi: 10.1016/j.cub.2015.03.023 (PMC4446794; doi:10.1016/j.cub.2015.03.023)
Supplement: Document S2. Article plus Supplemental Information [file mmc6.pdf]

Report

Current Biology

Genetic Dissection of a Regionally Differentiated Network for Exploratory Behavior in *Drosophila* Larvae

Graphical Abstract

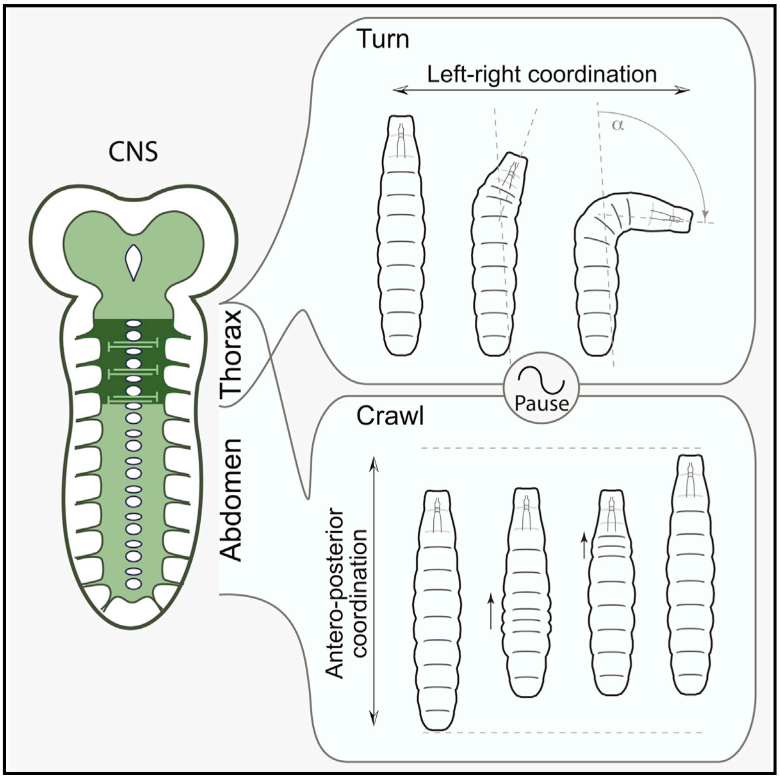

Highlights

- Exploration requires three neural functions: crawls, turns, and a transition switch
- Thoracic segments generate the asymmetric activity required for turns
- Crawls rely on symmetrical output from the thoracic and abdominal network
- Periodic pauses determine the frequency of transition between crawls and turns

Authors

Jimena Berni

Correspondence

jb672@cam.ac.uk

In Brief

By genetically dissecting the neural circuitry of exploratory behavior in *Drosophila* larvae, Berni shows that the three elements of the routine, namely the crawl, the pause, and the turn, are separable and distinct and that each can be definitively assigned to different parts of a regionally specialized thoracic/abdominal neuronal network.

Berni, 2015, Current Biology *25*, 1319–1326
May 18, 2015 ©2015 The Authors
http://dx.doi.org/10.1016/j.cub.2015.03.023

Current Biology
Report

Genetic Dissection of a Regionally Differentiated Network for Exploratory Behavior in *Drosophila* Larvae

Jimena Berni[1,*]
[1]Department of Zoology, University of Cambridge, Downing Street, CB2 3EJ Cambridge, UK
*Correspondence: jb672@cam.ac.uk
http://dx.doi.org/10.1016/j.cub.2015.03.023
This is an open access article under the CC BY license (http://creativecommons.org/licenses/by/4.0/).

SUMMARY

An efficient strategy to explore the environment for available resources involves the execution of random walks where straight line locomotion alternates with changes of direction. This strategy is highly conserved in the animal kingdom, from zooplankton to human hunter-gatherers [1–8]. *Drosophila* larvae execute a routine of this kind, performing straight line crawling interrupted at intervals by pause turns that halt crawling and redirect the trajectory of movement [9–11]. The execution of this routine depends solely on the activity of networks located in the thoracic and abdominal segments of the nervous system, while descending input from the brain serves to modify it in a context-dependent fashion [9]. I used a genetic method to investigate the location and function of the circuitry required for the different elements of exploratory crawling. By using the Slit-Robo axon guidance pathway to target neuronal midline crossing defects selectively to particular regions of the thoracic and abdominal networks, it has been possible to define at least three functions required for the performance of the exploratory routine: (1) symmetrical outputs in thoracic and abdominal segments that generate the crawls; (2) asymmetrical output that is uniquely initiated in the thoracic segments and generates the turns; and (3) an intermittent interruption to crawling that determines the time-dependent transition between crawls and turns.

RESULTS

Mutations in the gene *roundabout* (*robo*), coding for the receptor for the midline repellent Slit, cause axons and dendrites that will normally project on their own side of the CNS to cross the midline. This aberrant connectivity both of excitatory and inhibitory neurons leads to lethality at embryonic or larval stages (Figures 1A and S1; [10–12]). Interestingly, however, a high percentage of embryos with complete loss of function in the *robo* gene (*robo*^{1}/*robo*^{2} and *robo*^{2}/*robo*^{8}) hatch, and these animals can be used for behavioral analysis (Figure 1 and [10–12]).

In wild-type larvae, exploratory behavior consists of straight crawls, called runs, interrupted by pause turns [9, 13, 14]. The alternation between the two patterns of movements can be seen in the characteristic tracks left by wild-type newly hatched first instar larvae (Figures 2A and 2G; Movie S1). *robo*^{1}/+, *robo*^{2}/+, and *robo*^{8}/+ heterozygote controls execute the same routine and produce the same pattern of tracks (Figures 2D–2F). On the other hand, *robo* mutants have abnormal exploratory behavior. Their tracks show that they are fully capable of performing extended forward crawls but that these runs follow a circular path without sharp redirections generated by pause turns, and as a consequence, *robo* mutant larvae remain within a limited region of the available substrate (Figures 2B, 2C, and 2H; Movie S2).

I quantified the crawling abilities of wild-type and heterozygous *robo* larvae and compared them with the crawling of larvae with mutant allelic combinations of *robo* (*robo*^{1}/*robo*^{2} and *robo*^{2}/*robo*^{8}) by evaluating the denticle band movements (Figures 2I and S2). Wild-type and *robo* heterozygous larvae make equivalent numbers of forward waves of peristaltic contraction, but the *robo* mutants perform significantly less. In contrast, *robo* mutant larvae generate more backward waves (Figure 2I), although the total number of waves (forward and backward) is still significantly lower than controls. Notably, many *robo* mutant larvae have a postural deficit that causes them to lie on their sides rather than on their ventral surface with the consequence that the body is thrown into a curve generating circular paths instead of straight or slightly curved ones as observed during crawls in control larvae (Figures S2B and S2C). The duration of 96% of forward peristaltic waves in *robo*^{1}/*robo*^{2} and 95% in *robo*^{2}/*robo*^{8} mutant was the same as in wild-type animals (Figure 2K), although the distribution of the average duration per animal was only significantly different for *robo*^{1}/*robo*^{2} null larvae (Figure 2J). Thus, even though the growth of axons and dendrites across the midline is highly abnormal in *robo* mutants, they are capable of generating waves of coordinated peristaltic crawling, and the execution of these forward waves is largely indistinguishable from those seen in controls.

Next, I evaluated the performance of pause turns. In *robo*^{1}/*robo*^{2} and *robo*^{2}/*robo*^{8}, the number of pause turns is severely decreased, with a median value of zero indicating profound impairment (Figure 2L). This almost complete absence of pause turns (73% of the larvae analyzed for both genotypes never turned) is accompanied by a significant increase in the frequency

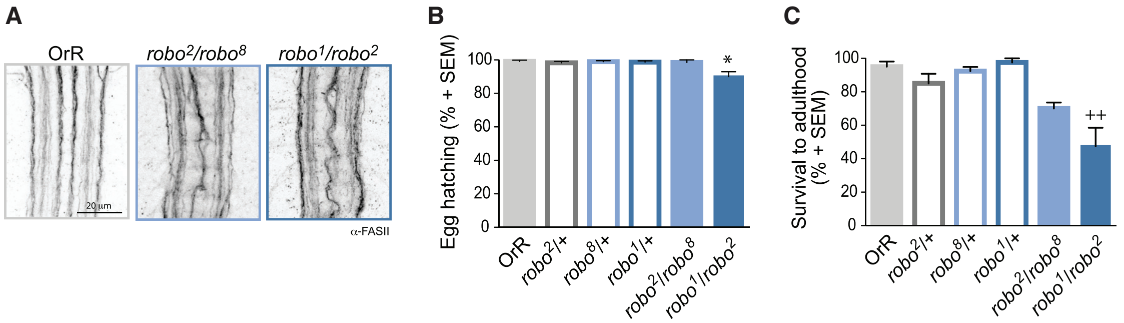

Figure 1. Survival of *Drosophila robo* Mutants

(A) Staining showing the Fasciclin II (FASII) positive axon tracts in first instar larval nerve cords for the different allelic combinations used in the study. The increase in the number of axons that cross the midline produces the characteristic circular appearance around the commissures. The penetrance of this phenotype is complete ($robo^1/robo^2$ 82/82; $robo^2/robo^8$ 42/42 and [10]). See Figure S1 for evaluation of excitatory and inhibitory midline connectivity.

(B) Average percentage of fertilized eggs hatching (±SEM).

(C) Average percentage of hatched larvae that survived until emergence of the adult (±SEM).

A Kruskal-Wallis test with Dunn's multiple comparison comparing OrR with all genotypes and each allelic combination with their heterozygote alleles was performed. Asterisk (*) indicates $p < 0.05$ when compared to OrR. $^{++}p < 0.05$ when compared to $robo^1/+$.

of a movement similar to the previously described "rearing" behavior (Figures 2M and S2E; [9]).

The sequence of movement in rearing is very similar to a pause turn (Movie S3) with the significant difference that there is no unilateral contraction of muscles in the anterior segments producing a left- or right-hand turn (Figure S2E). Instead, during rearing, the larva pauses at the end of a forward wave of contraction and raises the anterior segments of the body as a consequence of a sequential and bilaterally symmetrical contraction of the thoracic segments. Then, as abdominal segment 1 (A1) contracts, the thoracic segments relax, and the anterior end of the animal is propelled downward to hit the substrate (Figure S2E; Movie S4). Larvae resume crawling after one or a series of such contraction and rearing movement cycles. The characteristic bilateral symmetry of the rearing phenotype suggests that the unilateral control of muscle contractions required to execute turns fails in *robo* mutants.

Interestingly, the intermittent interruption to crawling (the pause) occurs in all larvae irrespective of whether they are turning or rearing, and this indicates that the mechanism that underlies this periodic switch between patterns of movement is unaffected in *robo* mutants. To test this idea, I calculated the proportion of transitions defined as the number of turns plus rearings per number of waves (Figure 2N). There were no significant differences between controls and *robo* mutants. Thus, the probability of triggering a transition is not affected by the aberrations in midline crossing found in *robo* mutant larvae.

To show whether behavioral phenotypes of *robo* mutants arise from a defective output of the central pattern generators (CPGs) for exploration, independent of sensory input, I performed calcium imaging experiments on the isolated nervous system. The compact organization of the nervous system of the larva, where neuromeres are fused, lends itself to the simultaneous evaluation of spontaneous activity in all thoracic and abdominal segments. I used the OK371-Gal4 driver line for glutamatergic neurons [15] to target the genetically encoded calcium indicator UAS-Gcamp3 [16] to all motor neurons and quantified changes in signal intensity in a defined region of the neuropile at the level of the intersegmental nerve [17] on both sides of the nerve cord (Figures 3A–3C).

Forward and backward waves of calcium influx can readily be detected propagating along the abdominal and thoracic segments in nervous systems isolated from control animals (OK371-GAL4, +/+, UAS-GCamp3) (Figures 3D and 3H). These waves of activity are synchronous on both sides of the nervous system, and in a semi-intact preparation, they have been shown to coincide with the wave of muscular contraction [18], strongly suggesting that they are indeed equivalent to the output from the CPG for peristaltic waves. In the isolated nervous system, the intersegmental phases are the same as in crawling animals (Figure S3), while the speed of wave propagation is slower, as reported in semi-intact preparations [18, 19] and when sensory input was acutely removed in freely moving larvae [20], supporting the idea that the preparation is as healthy as possible.

In addition, bilaterally asymmetric patterns of calcium influx can be detected in the anterior segments of control larvae, and these appear to be equivalent to asymmetric activity associated with turns in intact animals (Figures 3D and S3). To quantify the asymmetric activity, I calculated the normal value of the difference in the signal intensity between the left and right sides for each segment (Figure 3F). A comparison of the values obtained during periods of asymmetric and symmetric activity in control CNSs shows that there is a significant difference in the thoracic T2 and T3 and abdominal A1 and A2 segments (Figure 3I), all of which are segments that contract unilaterally when a larva is turning.

Forward and backward waves of bilaterally symmetric calcium influx are also seen in nervous systems isolated from larvae mutant for *robo* at frequencies that are not significantly different from control nervous systems (Figures 3E and 3H).

In contrast to control nervous systems, however, the asymmetric periods are severely reduced in *robo* mutants, with only one nervous system showing an asymmetric period in the anterior segments (Figures 3E and 3G). At the same time, the number of symmetric periods initiated in the thoracic segments and propagating posteriorly as far as A5 is increased (Figure 3E).

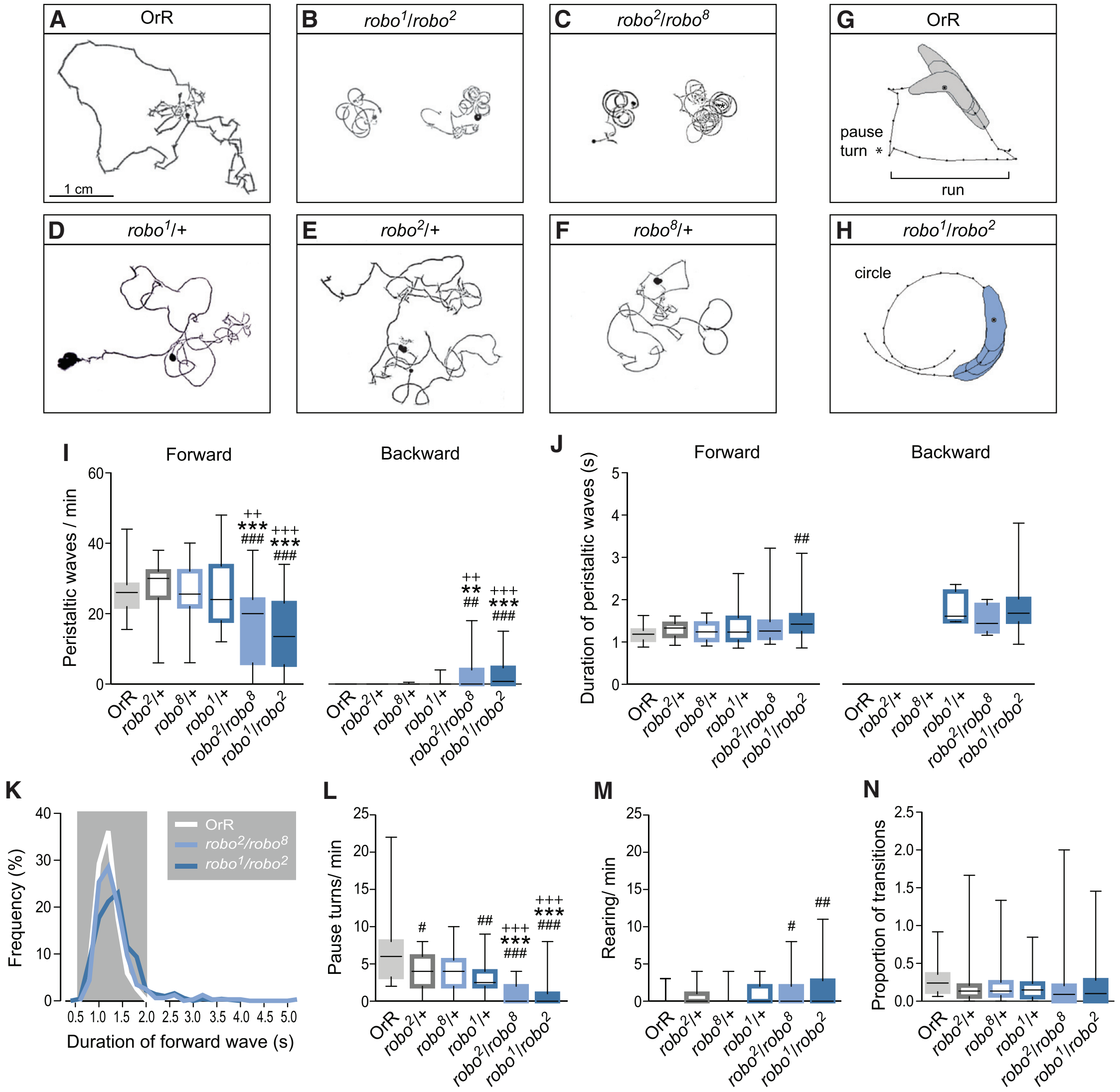

Figure 2. Locomotor Behavior of *robo* Mutant Larvae

(A–F) Characteristic tracks of first instar larvae. OrR (A) and heterozygous *robo* mutant alleles (D–F) explore by alternating straight movements with turns. *robo* mutant larvae perform circular crawls (B and C).

(G and H) Representative crawling patterns depicted by perimeter stacks. OrR larvae perform pause turns (G) (asterisk) by bending the anterior part of the body. *robo* mutants crawl in circles without performing turns (H). See also Movies S1, S2, S3, and S4 and Figure S2 for a detailed description of the behaviors.

(I) Number of forward and backward waves per minute.

(J) Duration of forward and backward waves in seconds.

(K) Distribution of duration of forward peristalsis for all waves analyzed. The gray box highlights the duration of waves in OrR larvae. Binning is 200 ms; OrR n = 375; *robo*^{1}/*robo*^{2} n = 471; *robo*^{2}/*robo*^{8} n = 319.

(L) Number of pause turns per minute.

(M) Number of rearing events per minute.

(N) Proportion of transitions. The number of pauses turns + rearing movements divided by the number of forward waves + backward waves was calculated. There are no significant differences between any genotype.

A Kruskal-Wallis test with Dunn's multiple comparison was used in (I), (J), (L), (M), and (N). Forward and backward waves were compared independently. Asterisk (*) indicates comparison with *robo*^{2}/+; + indicates comparison with the other heterozygote control; and # indicates comparison with OrR. *$p < 0.05$; **$p < 0.01$; ***$p < 0.001$; $^{+++}p < 0.001$. 32–41 larvae were evaluated per group. Boundaries of boxplots represent first and third quartiles; the middle line indicates the median. Whiskers indicate the highest and lowest value of each experimental group.

To quantify the symmetry of activity, I performed a comparison among segments of the difference in the signal intensity between the left and right sides in *robo* mutant nervous systems, confirming the lack of asymmetry in the output of the CPG for exploration (Figure 3J). A further comparison of the average of activity between *robo* mutants and control animals (calculated for the entire recording) highlighted the thoracic T2 and T3 segments as the region generating the major

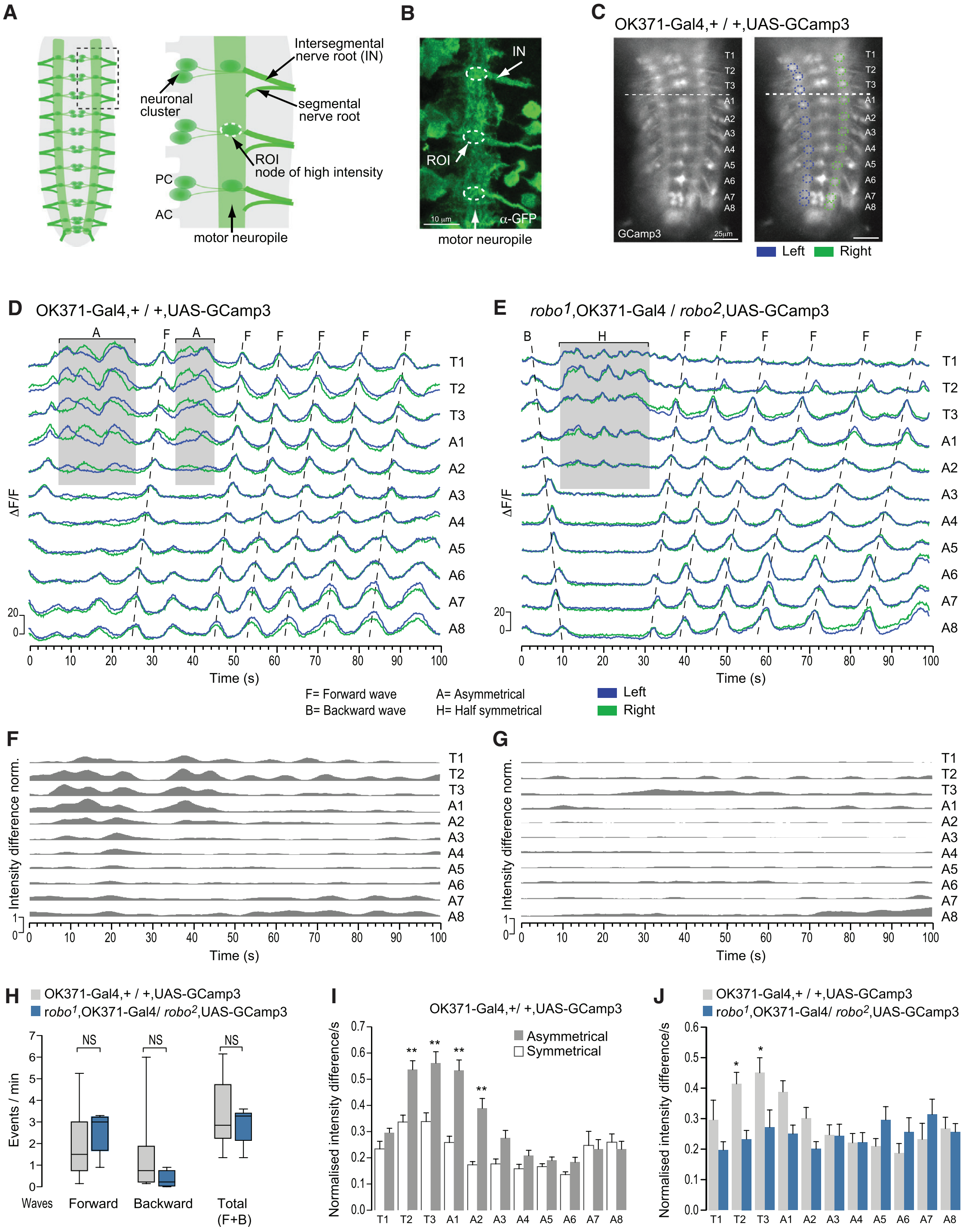

Figure 3. Output Activity of the CPG for Exploration

(A) Schematic denoting the location in the motor neuropile of the anatomically distinct nodes of fluorescence at the level of the intersegmental nerve that has been analyzed: ROI, region of interest; PC, posterior commissure; AC, anterior commissure.

(B) Equivalent region as in right panel of (A) in a CNS stained against GFP.

(C) Snapshot of GCaMP3 fluorescence in glutamatergic neurons in an isolated nerve cord. Right: the ROIs on both sides of the nerve cord are shown.

(D and E) Relative fluorescence change in isolated nervous systems. Left (blue) and right (green) sides of thoracic and abdominal segments were analyzed. Characteristic traces in control nervous system (D). Forward waves ("F") of activity propagating along segments can be observed as well as asymmetric periods ("A") in anterior segments. In $robo^2/robo^1$ mutants, forward and backward waves ("B") are present (E). The asymmetric periods are absent, but symmetrical periods in the half anterior segments ("H") can be observed.

(F and G) Normalized intensity difference between the left and right side for the recording shown in (D) and (E), respectively.

(legend continued on next page)

difference of asymmetry (see Figures 3J and S2C for a correlation analysis).

These experiments confirm that there is no asymmetry to the output from the CPG of *robo* loss-of-function mutants and highlight the differential requirement for appropriate connectivity across the midline for the generation of turns as opposed to straight line peristaltic crawling. They also point to the thoracic segments as a region of the nervous system that may be essential for the generation of a turn.

In order to define more precisely the region of the nervous system where appropriate midline crossing is indispensable for the generation of asymmetric outputs, I dissected the *robo* behavioral phenotype by analyzing the performance of larvae with progressively more normal patterns of connectivity along the antero-posterior axis of the nervous system. I took advantage of the existence of a regulator of Robo, *commissureless*, that sequesters the receptor before it reaches the membrane and thereby generates a cell-autonomous *robo* mutant phenotype in the targeted cells [21, 22].

Larvae that are UAS-*comm*; +/+; *tsh*-Gal4 (from now on *tsh*>*comm*) have normal patterns of connectivity in the brain lobes and subesophageal ganglion but have midline crossing defects in more posterior parts of the nerve cord, including all thoracic and abdominal segments (Figures 4A and 4B). The behavior of these larvae resembles that of *robo* mutant larvae (Figure 4). In particular, they crawl steadily and in a coordinated manner (Figures 4C and 4D), but the number of pause turns they make is significantly reduced, whereas rearing is increased compared to heterozygous controls (UAS-*comm*/+ and *tsh*-Gal4/+; Figures 4E–4G). The phenotype of *tsh*>*comm* animals is semi-penetrant; on average, they have the same defective performance of turns as both *robo* allelic combinations (non-significant differences between *tsh*>*comm*, $robo^2/robo^1$, and $robo^2/robo^8$ for rearings; Kruskal-Wallis and a Dunn's multiple comparison, and pause turns, ANOVA with post hoc analysis with Bonferroni correction).

A striking difference appears in the behavior of larvae in which normal connectivity extends from the brain lobes through the subesophageal and thorax segments with a midline phenotype that begins in and extends caudally from the posterior compartment of abdominal segment A1 to A7 (UAS-*comm*;; +/+;; *AbdA*-Gal4, also *AbdA*>*comm*; Figures 4A and 4B). These animals are now completely normal in their performance of pause turns (Figures 4H); their number is not significantly different from the two heterozygous controls (UAS-*comm*/+ and *AbdA*-Gal4/+) (Figure 4E). These animals also make very few rearing movements, and the number and duration of their forward and backward crawling waves are indistinguishable from those of the heterozygous controls (Figures 4C, 4D, and 4F).

In conclusion, these experiments define regional differences in a neuronal network for exploration. Posteriorly, bilaterally symmetric outputs required for a forward wave are initiated in the abdominal segments and pass forward to the thorax. More anteriorly, the asymmetric output required for unilateral contraction leading to a turn is initiated in the thorax and propagates to adjacent segments of the abdomen. While the propagation of bilaterally symmetric waves of contraction can proceed normally even if midline connectivity is disturbed, there is an absolute requirement for normal midline crossing for the asymmetric output of a turn, and it is this requirement that allows us to identify the thoracic segments of the network as the site of turn initiation.

DISCUSSION

In a previous paper, we showed that the exploratory crawling routine of the *Drosophila* larva is an intrinsic motor program, inherent to the thoracic and abdominal segments of the nervous system [9]. Using a genetic method, we were able to show that runs and pause turns continue normally if the brain and subesophageal ganglia are acutely silenced during exploratory crawling. The role of these more anterior segments of the nervous system is to modify the performance of the thoracic and abdominal routine in the presence of stimuli, for example, by altering the frequency and direction of turns when a food odor is detected [9, 26]. Here, I report the use of genetically targeted aberrations in axonal crossing at the midline to localize the regions of the nervous system that are essential for the facultative asymmetry in motor output that characterizes the turn as opposed to the symmetrical output of the straight crawl.

The first finding is that larvae that are null mutants for *robo* are capable of hatching and crawling over a substrate. Their crawling paths are unusually circular rather than straight, but this appears to be the effect of a postural deficit that causes the larvae to lie on their sides rather than on their ventral surfaces, with the consequence that the body is thrown into a curve, presumably by the differential strength of contraction in ventral as opposed to dorsal longitudinal muscles whose innervation is unaffected in *robo* mutants (Figure S4 and [27]). Despite this, the propagation of well-organized waves of muscle contraction from segment to segment proceeds normally during forward and backward crawls. Thus, the operation of the CPG for a straight crawl is unaffected by serious disruption to axonal crossing across the midline. The two sides of the animal are well coordinated, and this suggests that adequate and appropriate connections are maintained across the midline despite abnormal patterns of axonal growth. This is in stark contrast to the operation of the network required for turns. The performance of turns is

(H) Number of events per minute. n = 9 per group. A t test was performed comparing the number of waves between genotypes. Boxplots are described in the legend of Figure 2.

(I and J) Average normalized intensity per second (±SEM). The periods of symmetrical and asymmetrical activity are compared in control animals showing that differences in activity occur mainly in anterior segments (I). An ANOVA ($F_{21,375} = 18.36$; $p < 0.0001$) with Bonferroni's multiple comparison test comparing between genotypes for each segment was performed. **$p < 0.001$. n = 16 symmetrical periods and n = 20 asymmetrical periods. The difference of intensity for all active periods in control and *robo* mutants are compared (H). Anterior segments show a difference in symmetry. An ANOVA ($F_{21,161} = 2.967$; $p < 0.0001$) with Bonferroni's multiple comparison test comparing between genotypes for each segment was performed. *$p < 0.05$. In *robo* mutant, there is no significant difference among segments, indicating that the output of the CPG is symmetrical (Bonferroni's multiple comparison test comparing between segments). n = 8 per group.

See also Figure S3 for a comparison between neuronal activity recorded with calcium imaging and behavior.

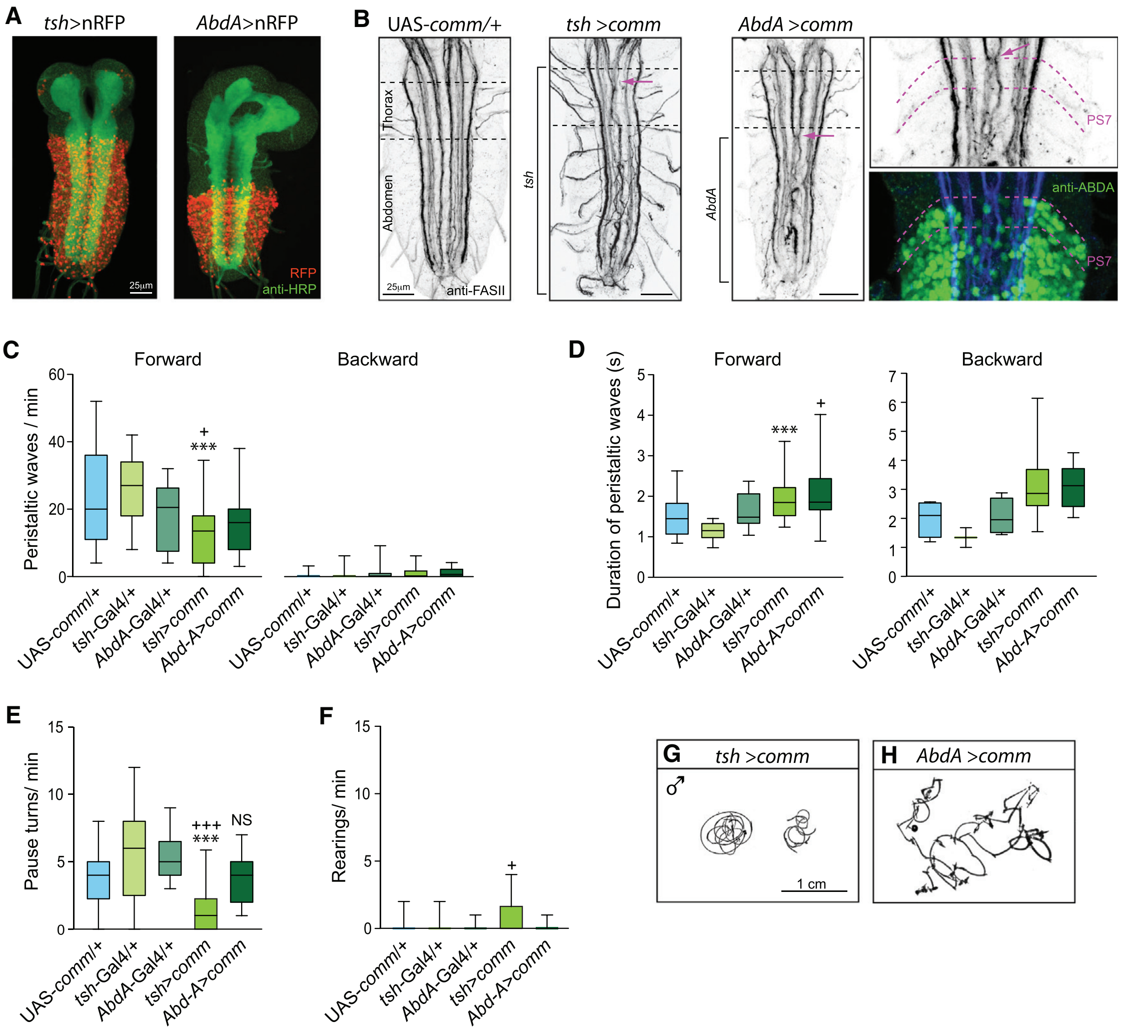

Figure 4. The Thoracic Segments Generate the Output for a Turn

(A) Pattern of expression of the Gal4 lines used. The driver lines were crossed to the fluorescent reporter UAS-nuclear red fluorescent protein (nRFP). Anti-HRP staining was used to show the neuropile. *tsh*-Gal4 is expressed in the thoracic and abdominal segments [11]. In *AbdA*-Gal4, the *gal4* is inserted in the largest intron of the *abd-A* transcription unit and reproduces the expression profile of Abd-A [23, 24] from the posterior half of A1 until the posterior half of A7 [25].

(B) Midline crossing defect as depicted by staining against Fas II. *tsh>comm* have midline crossing defects in thoracic and abdominal neuromeres, while aberrant crossing is only present in the abdominal segments of *AbdA>comm* larval nervous systems starting in segment A1, coinciding with the anterior boundary of AbdA expression (right panels). Magenta arrows indicate the anterior boundary of midline crossing defects.

(C) Number of forward and backward waves per minute.

(D) Duration of forward and backward waves in seconds.

(E) Number of pause turns per minute.

(F) Number of rearing events per minute.

(G and H) Representative tracks of male *tsh>comm* and *AbdA>comm* larvae.

A Kruskal-Wallis test with Dunn's multiple comparison was used. Asterisk (*) indicates comparison with the same heterozygous driver line. *$p < 0.05$; ***$p < 0.001$. + indicates comparison with UAS-*comm*/+. $^{+}p < 0.05$; $^{++}p < 0.01$; $^{+++}p < 0.001$. $n_{UAS\text{-}comm/+} = 23$; $n_{tsh\text{-}Gal4/+} = 23$; $n_{AbdA\text{-}Gal4/+} = 14$; $n_{tsh>comm} = 30$; $n_{AbdA>comm} = 19$. Boxplots are described in the legend of Figure 2.

completely abolished in the mutants, and this shows that, unlike the axial propagation of a wave, the generation of an asymmetric output depends absolutely on a normal pattern of connectivity across the midline. The simultaneous contraction of one side and relaxation of the other during a normal turn is likely to depend on the operation of reciprocal inhibitory connections across the midline [28] and one possible explanation is that as a consequence of the increased connectivity of both excitatory and inhibitory neurons across the midline (Figure S1), the strength of inhibition is decreased in comparison to excitatory connections in *robo* mutants. This notion is reinforced by the characteristic behavior that follows a pause in *robo* mutant larvae; contralateral inhibition is apparently overridden by symmetrical excitation so that the two sides of the animal contract together, causing the anterior segments to rear up, before relaxation and the resumption of a crawl.

The consequences of aberrant midline crossing for normally asymmetric motor outputs in the larva are reminiscent of the effects of induced defects in midline crossing seen in mice. In knockout mice for EphrinB3 or the EphA4 receptor tyrosine kinase, defective midline crossing of commissural interneurons [23, 29] causes synchronized activation of the normally reciprocating CPG for walking, with the result that the animals now exhibit a rabbit-like hopping phenotype, which is not unlike the rearing movement seen in *robo* mutant larvae. I find that an additional behavior in *Drosophila* larvae, which is likely to depend on reciprocal inhibition across the midline, namely self-righting from an inverted position, where asymmetric muscle contraction rotates the body, is also severely compromised in *robo* mutants (self-righting time: $robo^2/robo^1$ = 163 ± 23 s compared with $robo^2/+$ = 22 ± 7 s, $p < 0.001$, and with $robo^1/+$ = 36 ± 28 s, $p < 0.01$, Kruskal-Wallis test with Dunn's multiple comparisons). These behavioral findings are reinforced by the observations of spontaneous activity in the isolated nervous system where fictive crawling-like behavior is signaled by bilaterally symmetric waves of calcium influx propagated forward and backward along the thoracic and abdominal nervous system. These symmetric waves are complemented by episodic, asymmetric, unilateral activity, which is confined to the thorax and most anterior abdominal segments and may represent the fictive equivalent of a turn. In *robo* mutants, symmetric wave-like events continue unabated in the isolated nervous system, but asymmetric activity is completely abolished.

The observation that turns and putative "fictive" turns are restricted to the anterior-most segments of the animal and the isolated nervous system prompted me to try to identify the specific parts of the nervous system required to initiate this episodic, asymmetric redirection of exploratory crawling. Since I had found that the turn is uniquely sensitive to aberrant midline crossing, I decided to use a genetic method to target these aberrations to specific segments of the nervous system. My results show that when crossing is disrupted in the thorax and the abdomen turns are abolished. However, when crossing is normal in the thorax but disrupted in the abdomen, the larvae perform a normal exploratory routine of runs and pause turns. Thus, I conclude that, although anterior abdominal as well as thoracic segments contract asymmetrically during a turn, the initiation of this asymmetric event is thoracic and that this asymmetry is propagated posteriorly through a descending pathway as the movement progresses. Thus, the thoracic part of the thoracic and abdominal network appears to have special characteristics that enable the generation and propagation of an asymmetric motor output. I find it interesting that, while the turn is blocked in *robo* mutants, the episodic interruption to crawling, the pause, which precedes the redirection of movement in the wild-type, is not. Although propagated, wave-like output is a property of the entire thoracic and abdominal network and turns are a property of the thorax, it is not clear which part of the system is responsible for the pause, or whether it depends on a fluctuating property of the whole network, such as the level of excitation. Elucidating the neuronal substrate and mechanism responsible for the time-dependent transition between crawls and turns will be essential to understand the dispersion characteristics of the larva both during spontaneous exploration and in response to sensory stimuli [9, 26, 30–34].

EXPERIMENTAL PROCEDURES

Materials and methods can be found in the Supplemental Experimental Procedures.

SUPPLEMENTAL INFORMATION

Supplemental Information includes Supplemental Experimental Procedures, four figures, and four movies and can be found with this article online at http://dx.doi.org/10.1016/j.cub.2015.03.023.

ACKNOWLEDGMENTS

I thank Mike Bate, Matthias Landgraf, and Emiliano Merlo for constructive discussion and comments on the manuscript and Helen Skaer, Lidia Szczupak, Lorena Rela, Louise Couton, and Maarten Zwart for useful suggestions. I would like to express my deep gratitude to Mike Bate for his mentorship. I thank Nan Hu for his help with the embryo staining; Stefan Pulver and Tim Bayley for their assistance in the calcium imaging experiments; and Berthold Hedwig for lending the calcium imaging setup. I am grateful to Guy Tear, Samir Merabet, and the Bloomington Stock Center for fly stocks. I thank FlyBase for providing indispensable information and the Developmental Studies Hybridoma Bank for antibodies. J.B. was funded by EMBO long-term fellowship, by the Wellcome Trust Institutional Strategic Support Fund, by the Department of Zoology at University of Cambridge, and by a Sir Henry Dale Fellowship (Wellcome Trust and the Royal Society) Grant 105568/Z/14/Z. The work benefited from facilities supported by Wellcome Trust Equipment Grant WT079204.

Received: July 12, 2014
Revised: February 4, 2015
Accepted: March 16, 2015
Published: May 7, 2015

REFERENCES

1. Garcia, R., Moss, F., Nihongi, A., Strickler, J.R., Göller, S., Erdmann, U., Schimansky-Geier, L., and Sokolov, I.M. (2007). Optimal foraging by zooplankton within patches: the case of *Daphnia*. Math. Biosci. *207*, 165–188.
2. Pierce-Shimomura, J.T., Morse, T.M., and Lockery, S.R. (1999). The fundamental role of pirouettes in *Caenorhabditis elegans* chemotaxis. J. Neurosci. *19*, 9557–9569.
3. Hills, T., Brockie, P.J., and Maricq, A.V. (2004). Dopamine and glutamate control area-restricted search behavior in *Caenorhabditis elegans*. J. Neurosci. *24*, 1217–1225.
4. Reynolds, A.M., and Frye, M.A. (2007). Free-flight odor tracking in *Drosophila* is consistent with an optimal intermittent scale-free search. PLoS ONE *2*, e354.
5. McClellan, A.D., and Hagevik, A. (1997). Descending control of turning locomotor activity in larval lamprey: neurophysiology and computer modeling. J. Neurophysiol. *78*, 214–228.
6. Humphries, N.E., Queiroz, N., Dyer, J.R.M., Pade, N.G., Musyl, M.K., Schaefer, K.M., Fuller, D.W., Brunnschweiler, J.M., Doyle, T.K., Houghton, J.D.R., et al. (2010). Environmental context explains Lévy and Brownian movement patterns of marine predators. Nature *465*, 1066–1069.
7. Brown, C.T., Liebovitch, L.S., and Glendon, R. (2007). Lévy flights in Dobe Ju/'hoansi foraging patterns. Hum. Ecol. *35*, 129–138.
8. Viswanathan, G.M., Buldyrev, S.V., Havlin, S., da Luz, M.G., Raposo, E.P., and Stanley, H.E. (1999). Optimizing the success of random searches. Nature *401*, 911–914.
9. Berni, J., Pulver, S.R., Griffith, L.C., and Bate, M. (2012). Autonomous circuitry for substrate exploration in freely moving *Drosophila* larvae. Curr. Biol. *22*, 1861–1870.
10. Seeger, M., Tear, G., Ferres-Marco, D., and Goodman, C.S. (1993). Mutations affecting growth cone guidance in *Drosophila*: genes necessary for guidance toward or away from the midline. Neuron *10*, 409–426.

11. Kidd, T., Brose, K., Mitchell, K.J., Fetter, R.D., Tessier-Lavigne, M., Goodman, C.S., and Tear, G. (1998). *Roundabout* controls axon crossing of the CNS midline and defines a novel subfamily of evolutionarily conserved guidance receptors. Cell *92*, 205–215.
12. Spitzweck, B., Brankatschk, M., and Dickson, B.J. (2010). Distinct protein domains and expression patterns confer divergent axon guidance functions for *Drosophila* Robo receptors. Cell *140*, 409–420.
13. Green, C.H., Burnet, B., and Connolly, K.J. (1983). Organization and patterns of inter- and intraspecific variation in the behaviour of *Drosophila* larvae. Anim. Behav. *31*, 282–291.
14. Lahiri, S., Shen, K., Klein, M., Tang, A., Kane, E., Gershow, M., Garrity, P., and Samuel, A.D.T. (2011). Two alternating motor programs drive navigation in *Drosophila* larva. PLoS ONE *6*, e23180.
15. Mahr, A., and Aberle, H. (2006). The expression pattern of the *Drosophila* vesicular glutamate transporter: a marker protein for motoneurons and glutamatergic centers in the brain. Gene Expr. Patterns *6*, 299–309.
16. Tian, L., Hires, S.A., Mao, T., Huber, D., Chiappe, M.E., Chalasani, S.H., Petreanu, L., Akerboom, J., McKinney, S.A., Schreiter, E.R., et al. (2009). Imaging neural activity in worms, flies and mice with improved GCaMP calcium indicators. Nat. Methods *6*, 875–881.
17. Landgraf, M., Bossing, T., Technau, G.M., and Bate, M. (1997). The origin, location, and projections of the embryonic abdominal motorneurons of *Drosophila*. J. Neurosci. *17*, 9642–9655.
18. Kohsaka, H., Takasu, E., Morimoto, T., and Nose, A. (2014). A group of segmental premotor interneurons regulates the speed of axial locomotion in *Drosophila* larvae. Curr. Biol. *24*, 2632–2642.
19. Fox, L.E., Soll, D.R., and Wu, C.-F. (2006). Coordination and modulation of locomotion pattern generators in *Drosophila* larvae: effects of altered biogenic amine levels by the tyramine beta hydroxlyase mutation. J. Neurosci. *26*, 1486–1498.
20. Hughes, C.L., and Thomas, J.B. (2007). A sensory feedback circuit coordinates muscle activity in *Drosophila*. Mol. Cell. Neurosci. *35*, 383–396.
21. Keleman, K., Rajagopalan, S., Cleppien, D., Teis, D., Paiha, K., Huber, L.A., Technau, G.M., and Dickson, B.J. (2002). Comm sorts robo to control axon guidance at the *Drosophila* midline. Cell *110*, 415–427.
22. Mauss, A., Tripodi, M., Evers, J.F., and Landgraf, M. (2009). Midline signalling systems direct the formation of a neural map by dendritic targeting in the *Drosophila* motor system. PLoS Biol. *7*, e1000200.
23. Kullander, K., Butt, S.J.B., Lebret, J.M., Lundfald, L., Restrepo, C.E., Rydström, A., Klein, R., and Kiehn, O. (2003). Role of EphA4 and EphrinB3 in local neuronal circuits that control walking. Science *299*, 1889–1892.
24. Bender, W., and Hudson, A. (2000). P element homing to the *Drosophila* bithorax complex. Development *127*, 3981–3992.
25. Hirth, F., Hartmann, B., and Reichert, H. (1998). Homeotic gene action in embryonic brain development of *Drosophila*. Development *125*, 1579–1589.
26. Gomez-Marin, A., Stephens, G.J., and Louis, M. (2011). Active sampling and decision making in *Drosophila* chemotaxis. Nat. Commun. *2*, 441.
27. Wolf, B.D., and Chiba, A. (2000). Axon pathfinding proceeds normally despite disrupted growth cone decisions at CNS midline. Development *127*, 2001–2009.
28. Kiehn, O. (2011). Development and functional organization of spinal locomotor circuits. Curr. Opin. Neurobiol. *21*, 100–109.
29. Dottori, M., Hartley, L., Galea, M., Paxinos, G., Polizzotto, M., Kilpatrick, T., Bartlett, P.F., Murphy, M., Köntgen, F., and Boyd, A.W. (1998). EphA4 (Sek1) receptor tyrosine kinase is required for the development of the corticospinal tract. Proc. Natl. Acad. Sci. USA *95*, 13248–13253.
30. Luo, L., Gershow, M., Rosenzweig, M., Kang, K., Fang-Yen, C., Garrity, P.A., and Samuel, A.D.T. (2010). Navigational decision making in *Drosophila* thermotaxis. J. Neurosci. *30*, 4261–4272.
31. Louis, M., Huber, T., Benton, R., Sakmar, T.P., and Vosshall, L.B. (2008). Bilateral olfactory sensory input enhances chemotaxis behavior. Nat. Neurosci. *11*, 187–199.
32. Klein, M., Afonso, B., Vonner, A.J., Hernandez-Nunez, L., Berck, M., Tabone, C.J., Kane, E.A., Pieribone, V.A., Nitabach, M.N., Cardona, A., et al. (2015). Sensory determinants of behavioral dynamics in *Drosophila* thermotaxis. Proc. Natl. Acad. Sci. USA *112*, E220–E229.
33. Kane, E.A., Gershow, M., Afonso, B., Larderet, I., Klein, M., Carter, A.R., de Bivort, B.L., Sprecher, S.G., and Samuel, A.D.T. (2013). Sensorimotor structure of *Drosophila* larva phototaxis. Proc. Natl. Acad. Sci. USA *110*, E3868–E3877.
34. Godoy-Herrera, R., and Connolly, K. (2007). Organization of foraging behavior in larvae of cosmopolitan, widespread, and endemic *Drosophila* species. Behav. Genet. *37*, 595–603.

Current Biology
Supplemental Information

Genetic Dissection of a Regionally Differentiated Network for Exploratory Behavior in *Drosophila* Larvae

Jimena Berni

Supplemental Figures

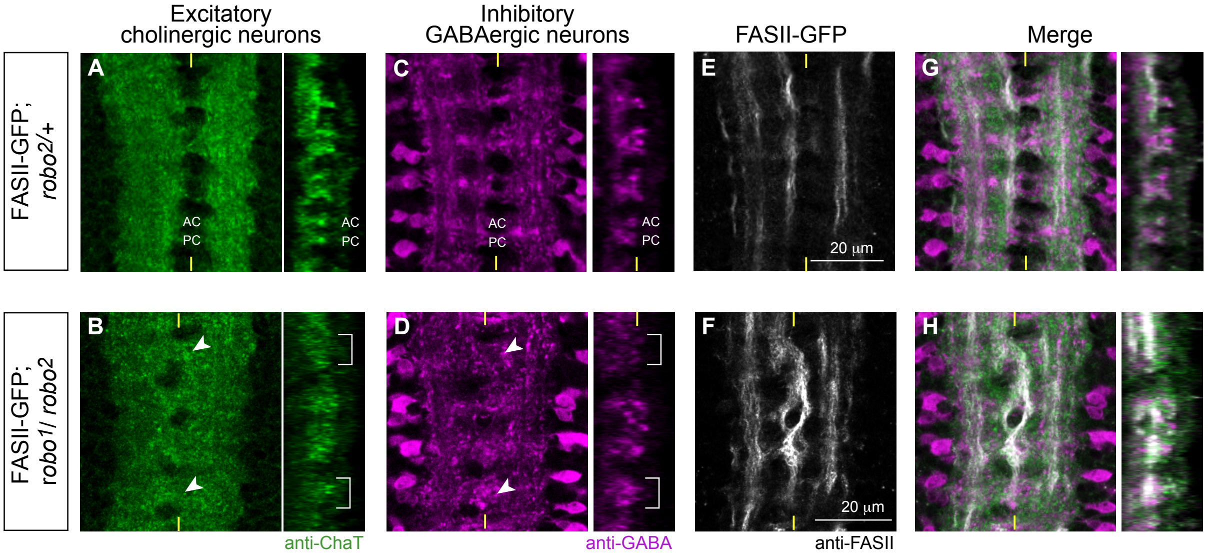

Figure S1 (related to Figure 1). Excitatory and Inhibitory Midline Connectivity is Increased in *robo* Null Mutants.

(A, B) Cholinergic excitatory neurons. In the control, FASII-GFP; *robo*^{2}/+, both the anterior commissures (AC) and posterior ones (PC) are clearly visible. In *robo*^{1}/ *robo*^{2} mutants the commissures are thicker (arrows and brackets show some examples) due to the increased number of excitatory axons crossing the midline.

(C, D) GABAergic inhibitory neurons. The commissures in *robo*^{1}/*robo*^{2} mutants are thickened as a consequence of increased inhibitory midline connectivity.

(E, F) FasII positive tracks for the XY slides shown, which is 1 μm thick.

(G,H) Merge images

Anterior is up. Yellow hash marks indicate the positions of the XZ section.

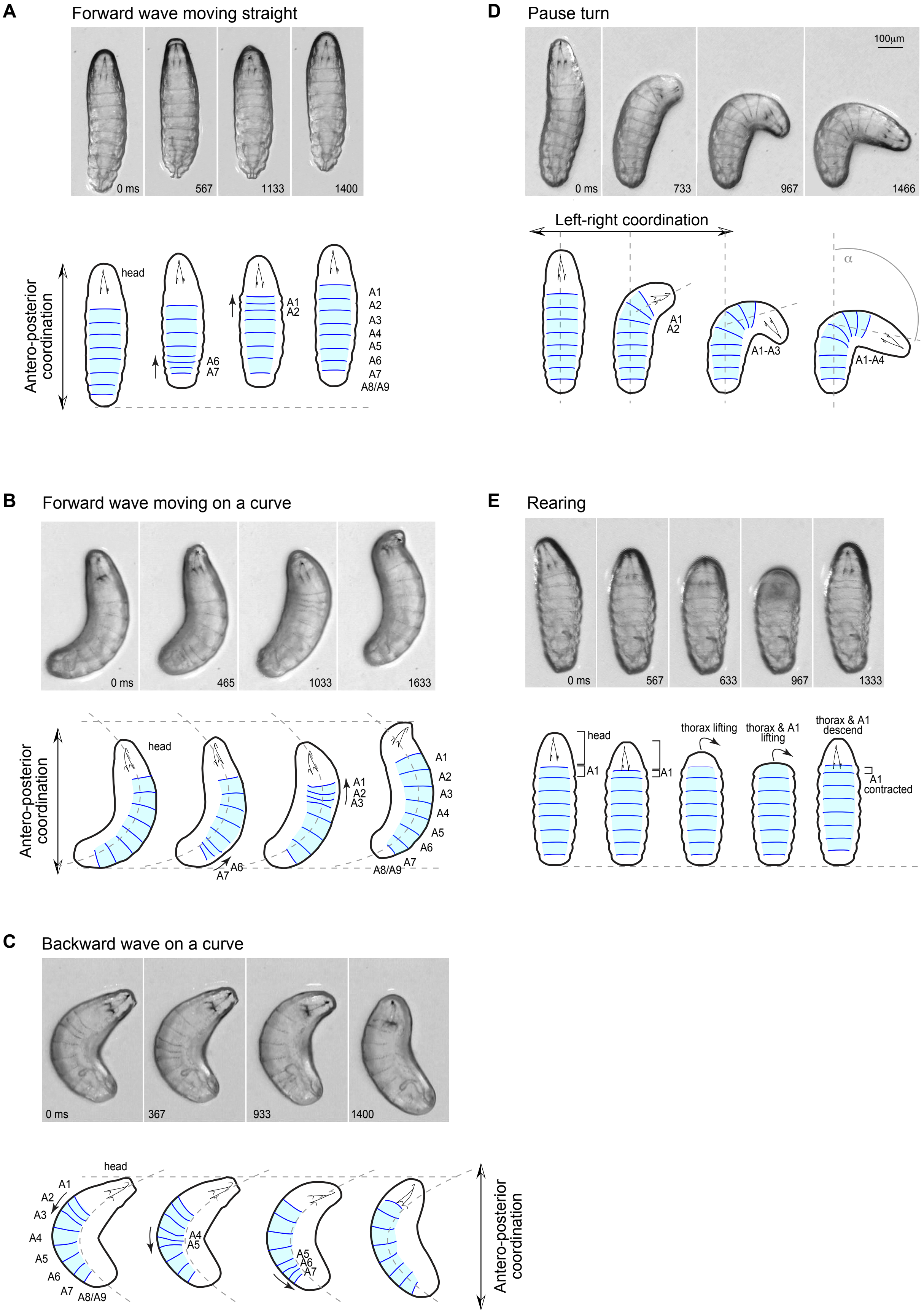
A
Forward wave moving straight
0 ms
567
1133
1400
Antero-posterior coordination
head
A1
A2
A3
A4
A5
A6
A7
A8/A9
B
Forward wave moving on a curve
0 ms
465
1033
1633
Antero-posterior coordination
head
A1
A2
A3
A4
A5
A6
A7
A8/A9
C
Backward wave on a curve
0 ms
367
933
1400
head
A1
A2
A3
A4
A5
A6
A7
A8/A9
Antero-posterior coordination
D
Pause turn
100μm
0 ms
733
967
1466
Left-right coordination
α
A1
A2
A1-A3
A1-A4
E
Rearing
0 ms
567
633
967
1333
head
A1
thorax lifting
thorax & A1 lifting
thorax & A1 descend
A1 contracted

Figure S2 (related to Figure 2). Description of Larval Patterns of Movement During Exploration.

Time-lapse of single peristaltic waves (A, B, C), a pause turn (D) and a rearing (E) with their corresponding diagrams below.

(A) Forward wave from a wild type. The larva advances in a straight line. Note that *robo* null mutant also perform forward waves in a straight line.

(B) Forward wave and backward wave (C) from a $robo^1/robo^2$ mutant lying on one side. The larva is thrown into a curve.

(D) A pause turn from a wild type larva

(E) Rearing behaviour of a $robo^1/robo^2$ mutant.

Arrows indicate the contracting abdominal segments at each time-point during a forward or backward peristaltic wave. The numbered abdominal segments in (B) correspond to the unilaterally contracting segments at each time-point during a turn. The light blue area indicates the denticle bands area in contact with the substrate.

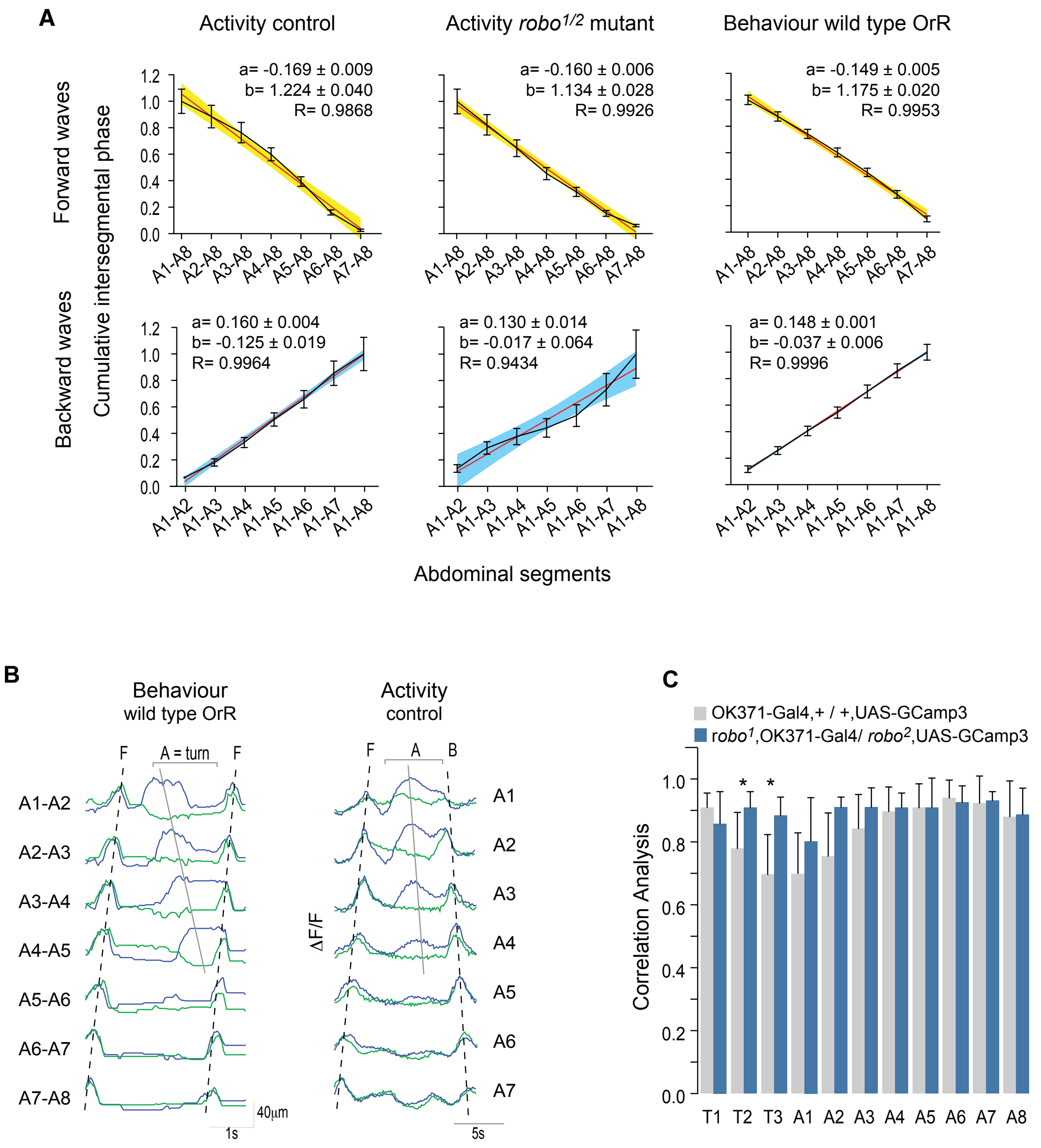

Figure S3 (related to Figure 3). Comparison between Neuronal Activity Recorded with Calcium Imaging and Behaviour.

(A) Comparison of intersegmental phases in isolated nervous system and crawling animals. Average cumulative intersegmental phase (+ SEM) in isolated nervous system and crawling larvae. The average phase of activity or contraction for each segment has been calculated respect to the beginning of the wave and is therefore refers to “cumulative”. Control larvae very rarely perform backward crawling. A gentle poke in the front of the animals was applied to stimulate them to do so. A linear regression analysis was performed, red line. The confidence intervals are highlighted in yellow and blue. a= slope; b= y value at x=0. The slopes of forward waves are not significantly different while the intercept at x=0 are (F =

9.03; DFn=2 DFd=17; P=0.002). There are no significant differences amongst backward waves.
Number of waves (Number of animals): Forward: control= 54 (9); *robo*$^{1/2}$= 66 (9); behaviour= 35 (15). Backward: control= 24 (9); *robo*$^{1/2}$= 10 (9); behaviour 14 (9).
(B) Comparison of the turning behaviour and asymmetrical activity in isolated nervous system. The temporal dynamics of segment activity and segment contraction during the asymmetrical period "A" are similar. A backward wave initiates only on one side and propagates posteriorly as far as A5.
The complete genotype of control animals is OK371-Gal4,+/ +,UAS-GCamp3 and of *robo*$^{1/2}$ animals is *robo*^{1}, OK371-Gal4 / *robo*^{2} ,UAS-GCamp3; F means forward wave and B means backward wave.
(C) A correlation analysis (average $\pm$ SEM) for the entire calcium imaging recordings was performed. Anterior segments T2 and T3 show a difference in symmetry amongst genotypes. An ANOVA comparing the r values between genotypes ($F_{21,161}$ = 2.386; p = 0.001) with post hoc comparison was performed. * p < 0.05. n = 8 per group.

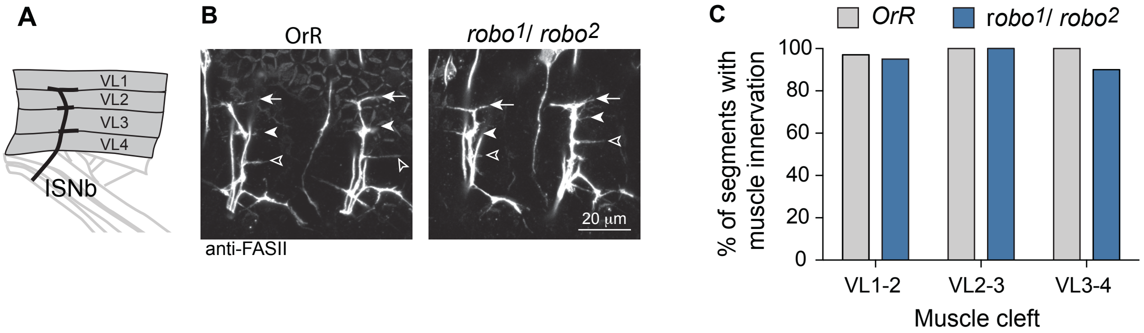

Figure S4 (related to the Discussion). The Innervation to the Ventral Longitudinal (VL) Muscles is Not Affected In *robo* Mutants.

(A) Projections of intersegmental nerve b (ISNb) to the embryonic ventral longitudinal muscles VL1 (12), VL2 (13), VL3 (6) and VL4 (7).

(B) ISNb motor neuron visualised with FasII antibody in stage 17. Arrows point to the VL1-2 innervation, filled arrowhead to VL2-3 and empty arrowhead to VL3-VL4.

(C) Percentage of segments showing innervation to VL muscles. An exact fisher test was performed comparing the wild type OrR with $robo^1$/ $robo^2$ mutants for each muscle cleft. VL1-2 P=1; VL2-3 P=1 and VL3-4 P=0.1153. 39 segments were analysed for each genotype.

Supplemental Experimental Procedures

Exploratory behaviour. Eggs were collected from flies kept on apple juice agar plates supplemented with yeast paste at 25°C.

Newly hatched first instar larvae were transferred to a 5cm petri dish coated with 0.9% agarose. The plate was inverted to view the denticle bands and 30 s – 2 min movies were captured at 30 fps with a JVC TKC1380 camera mounted on a Leica M420 microscope at 32X magnification. The wave duration was calculated as in Gjorgjieva *et al.* 2013 [S1]. The duration of a forward peristaltic wave was defined as the time between the first movement of the posterior end of the larva and the last movement of the anterior band of the first abdominal segment (A1). The reverse was used for backward waves (from A1 until the posterior end of the larva). The movements of thoracic segments were not included since their denticle bands are almost invisible and they move more independently. The timing of contraction was quantified with the open source software VCode 1.2.1 (http:// social.cs.uiuc.edu/projects/vcode.html). Turns were defined based on the detailed pattern of movements that can be evaluated by observing the denticle bands, they consist of an initial pause followed by the unilateral backward contraction initiated in thoracic segments and propagating backward until A4-A5. The different animals were analyzed blind.

The duration and number of waves or turns performed by each animal was averaged and represent one data, except in Figure 2K (see legend & results). At least 3 independent experiments were performed.

To image the tracks , newly hatched larvae were allowed to crawl for 3 hours at 25 degrees in the dark. The plates were inverted on top of an Ilford speed 3.1 m 100 photographic paper and illuminated for 4 seconds using an enlarger. Standard photographic method for development was used: 2 mins in Kodak Professional developer D19, rinse in water, 2 mins in Ilford Hypan rapid fixer, thorough wash in water. Black and white were then digitally inverted and background removed (technique developed by Michael Bate).

Dynamic Image Analysis (DIAS) software 3.4.2 (Sholl technologies, USA) was used to generate the perimeter stacks in Figures 2G and 2H.

Survival experiments. Hatching: adults were allowed to lay eggs overnight. The total number of larvae hatching from fertilized eggs was counted. Adult survival: 20 first instar larvae of each genotype were transferred and grown on apple juice agar plates supplemented with yeast paste. The number of adult emerging was counted. Experiments were repeated at least 3 times. Allelic combinations of *robo*^{1}, *robo*^{2} and *robo*^{8} were used to evaluate the *robo* loss of function in homozygosis while keeping in heterozygosis any other possible unspecific mutations present in the stocks that were generated with the mutagen ethyl methanesulfonate (EMS) [S2].

Calcium imaging. Experiments were performed on first instar larvae, 0– 4 h after hatching, at room temperature (23-25°C). Larvae were washed in H_2O and their nervous systems dissected in external saline for electrophysiology that consisted of: 135 mM NaCl, 5 mM KCl, 4 mM MgCl2.6H2O, 2 mM CaCl2, 5 mM N Tris (hydroxymethyl) methyl-2-aminoethanesulfonic acid and 36 mM sucrose, pH 7.15 [S3]. The isolated nervous systems were then stabilized by adhesion to a polylysine-coated cover slip glued to a 5 cm petridish filled with external saline. Preparations with a drift were excluded from the analysis.
A Leica DML-FS microscope with a 40x dipping objective was used for imaging. A 488 ± 15 nm wavelength light produced by a Cairn Optoscan Monochromator and passed through the epifluorescence port of the microscope was used to excite GCamp3. 2000 frames movies were captured using a cooled EMCCD Andor iXon DV887 camera at 5Hz at 512x512 pixel resolution using 2x pixel binning, and 2x frame binning through time. Data were transferred to Andor IQ software (version 1.9.1), recorded to a kinetic image disc.

Intensity information was extracted on ImageJ from regions of interest (ROIs). ROIs were defined as an oval enclosing the area of maximum intensity in the motor neuropile at the intersegmental nerve root [S4-S5] in abdominal segment one (A1). This was copied for all the other segments on the right and left side of the nerve cord. $\Delta F/F = (f_n - f_0)/f_0 * 100$ where f_0 is the average intensity.
Difference of Intensity between sides was calculated as: abs[ΔF/F left- ΔF/F right]. The area below curves filtered with a 2s window were calculated using spike 2. The average ΔF/F was normalized among experiments. Silent periods were not included in the average of normalized intensity. A correlation analysis was also performed using the entire recording.

The number of forward and backward events was empirically determined evaluating the $\Delta F/F$ curves along the abdominal segments.

Immunohistochemistry. Nervous systems of newly hatched first instar larvae were dissected in PB (100mM NaH_2PO_4/Na_2HPO_4) pH 7.2, transferred to a polylysine-coated cover slip fixed with 4% formaldehyde in PB for 20 min at room temperature (RT) and rinsed in PBS plus 0.3 % Triton X-100 (PBT) 3 X 15 min. Specimens were then incubated with anti-Fas II ID4 1/10; anti-ChaT 4B1 1/20 (Developmental Studies Hybridoma Bank, USA); rabbit anti-GABA A2052 1/2000 (Sigma) or chicken anti-GFP 1/2000 (Abcam) in PBT overnight at 4°C in a wet chamber, washed in PBT 4 X 15 min, and incubated with secondary antibodies at 1/500 in PBT for 3 h at RT: Alexa568 anti-Mouse; CF633 anti-rabbit; Alexa488 anti-Chicken (Invitrogen) and fluorescence-conjugated anti-HRP (Jackson Immuno Labs) at 1:50. Secondary antibodies were washed 4 X 30 min in PBT and specimens were mounted in Vectashield (Vector Laboratories) between two aluminium-foil spacers, to avoid distortion of nerve cords, under number 1 cover glasses. Image stacks were captured on a Leica TCS-SP-5 confocal microscope.

Embryo fixation and staining were performed as described in Patel 1994 [S6], anti-Fas II ID4 1/5.

Fly stocks. The following fly lines were provided by Bloomington Stock Center: UAS-stinger-RFP and Oregon-R. OK371-Gal4 is expressed in glutamatergic neurons, which includes all motor neurons and a subset of interneurons [S7]. UAS-*commissureless* on X [S8]. *AbdA*-gal4 was a present of Samir Merabet [S9]. $robo^{1}$, $robo^{2}$, $robo^{8}$ were kindly shared with me by Guy Tear. *tsh*-Gal4 was described in [S10]

Statistical Analysis. Statistical analysis was performed employing the Prism Graphpad 5.0b software package (2009). Normality was tested with a Kolmogorov-Smirnov test with Dallal-Wilkinson-Lillie for *p* value. A Bartlett's test for equal variances was also performed before deciding if a parametric or non-parametric test was going to be used to analyse the data.

Supplemental References

S1. Gjorgjieva, J., Berni, J., Evers, J. F., and Eglen, S. J. (2013). Neural circuits for peristaltic wave propagation in crawling *Drosophila* larvae: analysis and modeling. Front Comput Neurosci *7*, 24.

S2. Seeger, M., Tear, G., Ferres-Marco, D., and Goodman, C. S. (1993). Mutations affecting growth cone guidance in *Drosophila*: genes necessary for guidance toward or away from the midline. Neuron *10*, 409–426.

S3. Muraro, N. I., Weston, A. J., Gerber, A. P., Luschnig, S., Moffat, K. G., and Baines, R. A. (2008). Pumilio binds para mRNA and requires Nanos and Brat to regulate sodium current in *Drosophila* motoneurons. J Neurosci *28*, 2099–2109.

S4. Landgraf, M., Bossing, T., Technau, G., and Bate, M. (1997). The origin, location, and projections of the embryonic abdominal motorneurons of *Drosophila*. J Neurosci *17*, 9642–9655.

S5. Kohsaka, H., Takasu, E., Morimoto, T., and Nose, A. (2014). A group of segmental premotor interneurons regulates the speed of axial locomotion in *Drosophila* larvae. Curr Biol *24*, 2632–2642.

S6. Patel, N. H. (1994). Imaging neuronal subsets and other cell types in whole-mount *Drosophila* embryos and larvae using antibody probes. Methods Cell Biol. *44*, 445–487.

S7. Mahr, A., and Aberle, H. (2006). The expression pattern of the *Drosophila* vesicular glutamate transporter: a marker protein for motoneurons and glutamatergic centers in the brain. Gene Expr Patterns *6*, 299–309.

S8. Kidd, T., Russell, C., Goodman, C. S., and Tear, G. (1998). Dosage-sensitive and complementary functions of *roundabout* and *commissureless* control axon crossing of the CNS midline. Neuron *20*, 25–33.

S9. Hudry, B., Viala, S., Graba, Y., and Merabet, S. (2011). Visualization of protein interactions in living *Drosophila* embryos by the bimolecularfluorescence complementation assay. BMC biology *9*, 5.

S10. Fasano, L., Röder, L., Coré, N., Alexandre, E., Vola, C., Jacq, B., and Kerridge, S. (1991). The gene teashirt is required for the development of *Drosophila* embryonic trunk segments and encodes a protein with widely spaced zinc finger motifs. Cell *64*, 63–79.
